# PRC1 Catalytic Activity Is Central to Polycomb System Function

**DOI:** 10.1016/j.molcel.2019.12.001

**Published:** 2020-02-20

**Authors:** Neil P. Blackledge, Nadezda A. Fursova, Jessica R. Kelley, Miles K. Huseyin, Angelika Feldmann, Robert J. Klose

**Affiliations:** 1 Department of Biochemistry, University of Oxford, South Parks Rd., Oxford OX1 3QU, UK

**Keywords:** polycomb, chromatin, gene expression, transcription, PRC1, H2AK119ub1, histone modification, conditional point mutant

## Abstract

The Polycomb repressive system is an essential chromatin-based regulator of gene expression. Despite being extensively studied, how the Polycomb system selects its target genes is poorly understood, and whether its histone-modifying activities are required for transcriptional repression remains controversial. Here, we directly test the requirement for PRC1 catalytic activity in Polycomb system function. To achieve this, we develop a conditional mutation system in embryonic stem cells that completely removes PRC1 catalytic activity. Using this system, we demonstrate that catalysis by PRC1 drives Polycomb chromatin domain formation and long-range chromatin interactions. Furthermore, we show that variant PRC1 complexes with DNA-binding activities occupy target sites independently of PRC1 catalytic activity, providing a putative mechanism for Polycomb target site selection. Finally, we discover that Polycomb-mediated gene repression requires PRC1 catalytic activity. Together these discoveries provide compelling evidence that PRC1 catalysis is central to Polycomb system function and gene regulation.

## Highlights

• PRC1 catalysis drives PRC2 occupancy and H3K27me3 deposition at target sites
• cPRC1 binding and PRC1-mediated chromatin interactions require PRC1 catalysis
• DNA-binding vPRC1 complexes occupy target sites independently of PRC1 catalysis
• PRC1 catalytic activity is essential for Polycomb-mediated gene repression

## Graphical Abstract

**Figure:**
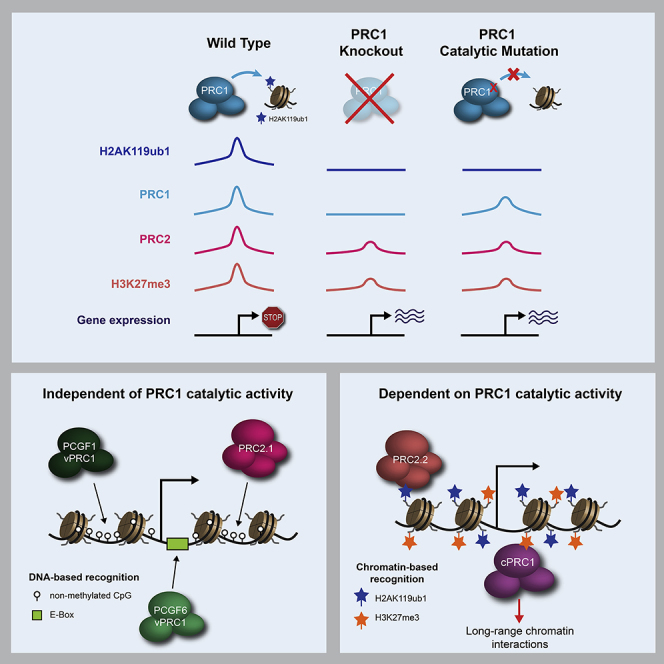

## Teaser

In this study, Blackledge et al. generate a conditional catalytic point mutant system to test the contribution of PRC1 catalytic activity to the Polycomb repressive system. They reveal that PRC1 catalytic activity is essential for Polycomb chromatin domain formation, long-range chromatin interactions between Polycomb target sites, and Polycomb-mediated gene repression.

## Introduction

Eukaryotic DNA is wrapped around histone octamers to form nucleosomes and chromatin, which organize DNA within the confines of the nucleus. In addition to this essential packaging role, histones are also post-translationally modified, and this is proposed to regulate important chromatin-based processes (Atlasi and Stunnenberg, 2017, Kouzarides, 2007, Musselman et al., 2012b). For example, removal of enzymes that modify histones around gene promoters can lead to alterations in gene expression (Bannister and Kouzarides, 2011). However, these enzymes often contain multiple conserved domains, some of which are not required for catalysis, and typically form large multi-protein complexes (DesJarlais and Tummino, 2016, Schuettengruber et al., 2017). This has made it challenging to understand the extent to which the catalytic activity of histone modifying enzymes contributes to nuclear processes such as transcription.

The Polycomb repressive system is an essential regulator of developmental gene expression (reviewed in Blackledge et al., 2015, Di Croce and Helin, 2013, Schuettengruber et al., 2017). Polycomb group (PcG) proteins typically belong to one of two multi-protein complexes that have chromatin-modifying activity and repress transcription: Polycomb Repressive Complex 1 (PRC1) is an E3 ubiquitin ligase that mono-ubiquitylates histone H2A at lysine 119 (H2AK119ub1) (de Napoles et al., 2004, Wang et al., 2004a), and Polycomb Repressive Complex 2 (PRC2) is a methyltransferase that mono-, di-, and tri-methylates histone H3 at lysine 27 (H3K27me1, H3K27me2, and H3K27me3) (Cao et al., 2002, Czermin et al., 2002, Kuzmichev et al., 2002, Müller et al., 2002). In vertebrates, PRC1 and PRC2 can recognize target gene promoters associated with CpG islands (CGIs) and form Polycomb chromatin domains that are characterized by high-level enrichment of these complexes and the histone modifications that they deposit (Mikkelsen et al., 2007). Perturbations of Polycomb repressive complexes can lead to alterations in the levels of H2AK119ub1 and H3K27me3 and inappropriate expression of Polycomb target genes (Boyer et al., 2006, Endoh et al., 2008, Endoh et al., 2017, Fursova et al., 2019, Leeb et al., 2010, Pasini et al., 2007, Rose et al., 2016). In turn, these molecular pathologies can cause developmental abnormalities and other human disease states (Pasini and Di Croce, 2016, Poynter and Kadoch, 2016, Richly et al., 2011).

The catalytic core of PRC1 is formed by RING1A or RING1B and one of six PCGF proteins, giving rise to an array of biochemically distinct canonical PRC1 (cPRC1) or variant PRC1 (vPRC1) complexes (Gao et al., 2012, Hauri et al., 2016, Wang et al., 2004a). cPRC1 complexes assemble around PCGF2/4 and bind to chromatin via a CBX subunit that recognizes H3K27me3 (Bernstein et al., 2006, Cao et al., 2002, Wang et al., 2004b). vPRC1 complexes, which can utilize all six PCGFs, incorporate RYBP/YAF2 in place of CBX proteins and do not bind H3K27me3 (Gao et al., 2012, Morey et al., 2013, Tavares et al., 2012). The recruitment of vPRC1 complexes to target sites relies, at least in part, on their DNA-binding activities: PCGF1-vPRC1 associates with KDM2B that recognizes CGIs (Farcas et al., 2012, He et al., 2013, Wu et al., 2013), and PCGF6-vPRC1 associates with MGA/MAX DNA-binding factors (Endoh et al., 2017, Scelfo et al., 2019, Stielow et al., 2018). vPRC1 complexes containing PCGF1/3/5/6 are highly active at catalyzing H2AK119ub1 *in vitro* and deposit the majority of H2AK119ub1 *in vivo*, whereas cPRC1 contributes little to this process (Fursova et al., 2019, Rose et al., 2016, Taherbhoy et al., 2015). The core of PRC2 is composed of EZH1/2, EED, SUZ12, and RBAP46/48 (Cao et al., 2002, Czermin et al., 2002, Kuzmichev et al., 2002). PRC2 complexes are subdivided into PRC2.1 and PRC2.2 based on their accessory subunits. PRC2.1 associates with PCL1/2/3, which bind CpG-rich DNA (Li et al., 2017, Perino et al., 2018) and histone H3 lysine 36 tri-methylation (Ballaré et al., 2012, Brien et al., 2012, Cai et al., 2013, Musselman et al., 2012a). In addition, the EPOP and PALI1/2 subunits of PRC2.1 are thought to fine-tune PRC2.1-dependent gene repression (Beringer et al., 2016, Conway et al., 2018, Liefke et al., 2017). In contrast, PRC2.2 associates with AEBP2 and JARID2, with JARID2 recognizing H2AK119ub1 deposited by PRC1 (Cooper et al., 2016, Kalb et al., 2014).

Early studies dissecting how Polycomb chromatin domains form have proposed a PRC2-initiated hierarchical mechanism whereby PRC2 recruitment to target genes via transcription factors or non-coding RNAs leads to H3K27me3 deposition and subsequent binding of cPRC1 complexes (Cao et al., 2002, Min et al., 2003, Wang et al., 2004b). The self-polymerizing PHC subunits (PHC1/2/3) in cPRC1 complexes can compact chromatin and create long-range interactions between Polycomb target sites, and these H2AK119ub1-independent mechanisms were proposed to underpin Polycomb-mediated gene repression (Eskeland et al., 2010, Francis et al., 2004, Grau et al., 2011, Isono et al., 2013, Kim et al., 2002, Kundu et al., 2017). However, recent work has shown that a PRC2-initiated pathway is not sufficient to explain Polycomb-mediated gene repression, as mouse embryonic stem cells (ESCs) lacking PRC2 or cPRC1 complexes show minimal derepression of Polycomb target genes (Fursova et al., 2019, Riising et al., 2014). In contrast, removal of vPRC1 complexes caused a near-complete loss of H2AK119ub1, erosion of Polycomb chromatin domains, and widespread reactivation of Polycomb target genes (Fursova et al., 2019). These observations suggest that vPRC1 complexes may contribute centrally to Polycomb chromatin domain formation and gene repression via their capacity to deposit H2AK119ub1.

Attempts to dissect the importance of PRC1 catalysis in mammals have yielded conflicting outcomes. RING1B catalytic activity was reported to be non-essential for PRC1-mediated gene repression in mouse ESCs and for early mouse development (Eskeland et al., 2010, Illingworth et al., 2015). However, in these experiments, RING1A-containing PRC1 complexes that can also deposit H2AK119ub1 (Endoh et al., 2012, Leeb et al., 2010) were intact. When RING1B catalysis was disrupted in RING1A null cells, PRC1-mediated gene repression was affected, but less dramatically than by complete removal of RING1A/B (Cohen et al., 2018, Endoh et al., 2012). In these studies, PRC1 catalytic activity was perturbed by substituting isoleucine 53 for alanine in RING1B (I53A) to disrupt the interaction between RING1B and its E2 ubiquitin-conjugating enzyme. However, earlier *in vitro* biochemical analysis suggested that RING1B^I53A^ is hypomorphic (Buchwald et al., 2006), and more recent studies have demonstrated that this mutation is not sufficient to eliminate H2AK119ub1 *in vivo* (Kundu et al., 2017, Tsuboi et al., 2018). In contrast, a double mutation combining I53A and a substitution of glutamic acid 56 to lysine (D56K) appeared to render RING1B catalytically inactive (Tsuboi et al., 2018). Using this double mutation and a neuronal cell fate restriction model, PRC1 catalysis was suggested to play an essential role in gene repression during early neurogenesis but become less important at later stages of development. However, given the small number of genes analyzed in this study, the generality of these conclusions remains to be tested. Therefore, the extent to which PRC1 catalytic activity is required for Polycomb system function remains poorly understood and a point of active debate (Blackledge et al., 2015, Schuettengruber et al., 2017, Simon and Kingston, 2009, Steffen and Ringrose, 2014).

Here, we systematically dissect the role of PRC1 catalytic activity in Polycomb-mediated gene regulation. By developing a new conditional PRC1 catalytic point mutant system in ESCs, we discover that PRC1 catalysis drives PRC2 binding and H3K27me3 at Polycomb target sites. This is necessary for occupancy of cPRC1 complexes, which mediate long-range interactions between Polycomb chromatin domains. Furthermore, we show that variant PRC1 complexes with inherent DNA-binding activities localize to target sites independently of PRC1 catalytic activity, providing a putative mechanism for Polycomb target site selection. Finally, and most importantly, we discover that loss of PRC1 catalysis largely phenocopies the gene expression and cellular defects caused by complete removal of PRC1. Together, these discoveries reveal a fundamental requirement for the catalytic activity of PRC1 in gene repression and Polycomb system function in ESCs.

## Results

### RING1B^I53A/D56K^ Forms Catalytically Inactive PRC1 Complexes

**Figure 1:**
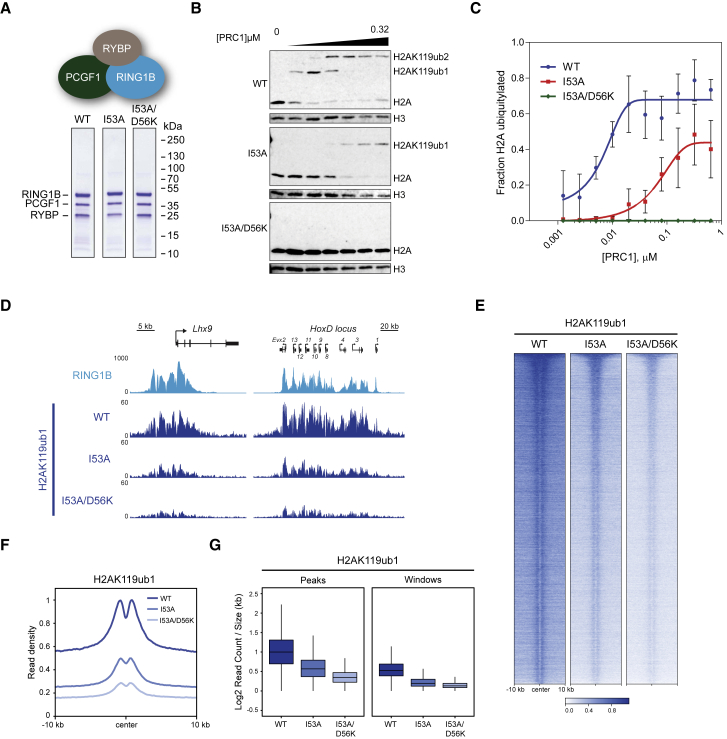
RING1B^I53A/D56K^ Is Catalytically Inactive but Not Sufficient to Eliminate H2AK119ub1 *In Vivo* (A) Coomassie-stained gels of affinity-purified RING1B-PCGF1-RYBP complexes. (B) *In vitro* E3 ubiquitin ligase assays in which conversion of unmodified histone H2A to ubiquitylated forms was measured by western blot with an H2A-specific antibody. An H3-specific antibody was used as a control. (C) Quantification of the mean fraction of histone H2A ubiquitylation across a range of PRC1 concentrations from *in vitro* E3 ubiquitin ligase assays in (B). Error bars show SEM (n = 2 or more). (D) Genomic snapshots of classical RING1B-bound loci, showing cChIP-seq for RING1B in wild-type ESCs and cChIP-seq for H2AK119ub1 in RING1B^WT^, RING1B^I53A^, and RING1B^I53A/D56K^ ESCs. (E) Heatmap analysis of H2AK119ub1 cChIP-seq at RING1B-bound sites in RING1B^WT^, RING1B^I53A^, and RING1B^I53A/D56K^ ESCs. Genomic regions were sorted based on RING1B occupancy in untreated PRC1^CKO^ ESCs. (F) Metaplot analysis of data shown in (E). (G) Boxplots comparing normalized H2AK119ub1 cChIP-seq signal at RING1B-bound sites and 100 kb genomic windows in RING1B^WT^, RING1B^I53A^, and RING1B^I53A/D56K^ ESCs. See also Figure S1.

In order to examine the contribution of PRC1 catalysis to Polycomb system function in cells, it was important to first identify a catalytic mutant of RING1B that was capable of forming PRC1 complexes yet completely lacked catalytic activity as validated by *in vitro* biochemical assays. RING1B^D56K^ had previously been shown to inactivate a minimal PRC1 catalytic core complex *in vitro* (Bentley et al., 2011, Taherbhoy et al., 2015), and, when combined with I53A, appeared to produce PRC1 complexes incapable of depositing H2AK119ub1 *in vivo* (Tsuboi et al., 2018). To ensure that RING1B^I53A/D56K^ inactivates PRC1 catalytic activity yet does not disrupt PRC1 complex formation, and to directly compare its activity to RING1B^I53A^, we reconstituted the highly active PCGF1/RING1B/RYBP vPRC1 complex with either wild-type RING1B, RING1B^I53A^, or RING1B^I53A/D56K^ (Figures 1A and S1A). Mutant forms of RING1B were efficiently incorporated into the complex, indicating that they do not disrupt formation of the PRC1 catalytic core (Figure 1A). We then compared the activity of these reconstituted complexes using *in vitro* ubiquitylation assays on recombinant nucleosome substrates (Figure 1B). In agreement with previous reports (Bentley et al., 2011, Buchwald et al., 2006), wild-type PRC1 ubiquitylated histone H2A efficiently, and RING1B^I53A^-containing PRC1 was less active but clearly retained catalytic activity (Figures 1B and 1C). Importantly, however, RING1B^I53A/D56K^-containing PRC1 produced no detectable H2A ubiquitylation (Figures 1B and 1C), indicating that RING1B^I53A/D56K^ completely disrupts PRC1 catalysis.

### RING1B^I53A^ and RING1B^I53A/D56K^ ESCs Retain Significant Levels of H2AK119ub1

To characterize how RING1B^I53A^ and RING1B^I53A/D56K^ affect H2AK119ub1 at the genome scale, we introduced the I53A or I53A/D56K mutations into the endogenous *Ring1b* gene in mouse ESCs (Figure S1B). Western blot analysis revealed that RING1B levels were similar in RING1B^WT^, RING1B^I53A^, and RING1B^I53A/D56K^ cells (Figure S1C), and co-immunoprecipitation experiments demonstrated that PRC1 complexes containing mutant RING1B formed normally (Figure S1D). Next, using calibrated chromatin immunoprecipitation sequencing (cChIP-seq), we found that in RING1B^I53A^ cells, H2AK119ub1 was reduced both at highly enriched RING1B-occupied sites (Figures 1D–1G) and in the low-level blanket throughout the genome (Figure 1G). Importantly, H2AK119ub1 levels were further diminished in RING1B^I53A/D56K^ cells (Figures 1D–1G), and this was also evident in western blot analysis of bulk histones (Figure S1C). These observations support the conclusion that RING1B^I53A^ is hypomorphic, in agreement with our *in vitro* ubiquitylation assays and previous studies in neural stem cells (Tsuboi et al., 2018). Importantly, these analyses also demonstrate that significant levels of H2AK119ub1 remain in RING1B^I53A^ ESCs previously used to test the requirement for PRC1 catalysis in Polycomb system function (Eskeland et al., 2010, Illingworth et al., 2015, Kundu et al., 2017). Furthermore, despite RING1B^I53A/D56K^ being catalytically inactive *in vitro*, RING1B^I53A/D56K^ cells retained some H2AK119ub1, consistent with RING1A contributing to deposition of H2AK119ub1 in ESCs (Endoh et al., 2012, Leeb et al., 2010). We therefore reasoned that complete inactivation of PRC1 catalysis and elimination of H2AK119ub1 would require double catalytic mutations being introduced into both RING1A and RING1B. Engineering the analogous mutations (I50A/D53K) into *Ring1a* was highly efficient, but attempts to combine these mutations with RING1B^I53A/D56K^ yielded no ESC lines. This suggests that PRC1 catalysis may be essential for ESC viability and Polycomb system function.

### A New Conditional Point Mutant System to Inactivate PRC1 Catalysis

**Figure 2:**
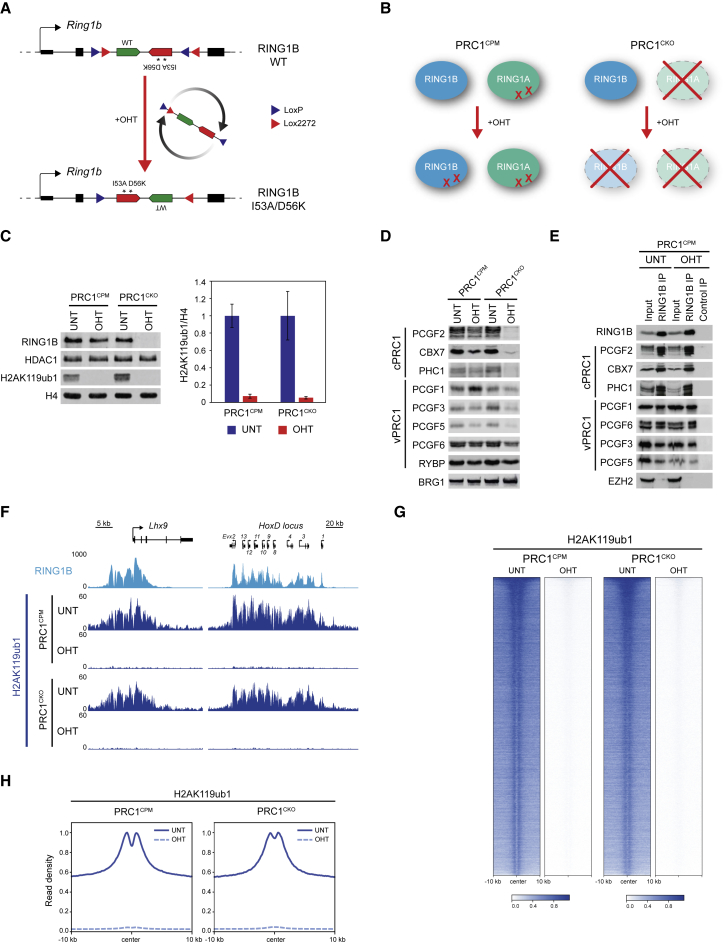
A Conditional Point Mutant System to Inactivate PRC1 Catalysis (A) A schematic of the engineered *Ring1b* locus in the PRC1^CPM^ system before and after OHT addition. (B) A schematic of the PRC1^CPM^ and PRC1^CKO^ ESCs. (C) Western blot analysis of RING1B (with HDAC1 as a loading control) and H2AK119ub1 (with H4 as a loading control) in untreated and OHT-treated PRC1^CPM^ and PRC1^CKO^ cells (left panel). Quantification of H2AK119ub1 levels relative to histone H4. Error bars show SEM (n = 4) (right panel). (D) Western blot analysis of cPRC1- and vPRC1-specific subunits in untreated and OHT-treated PRC1^CPM^ ESCs (with BRG1 as a loading control). (E) Immunoprecipitation of RING1B from untreated and OHT-treated PRC1^CPM^ ESCs followed by western blot for cPRC1 and vPRC1 components. Western blot for EZH2 (a PRC2 component) was used as a negative control. For OHT-treated PRC1^CPM^ ESCs, a control IP was performed with an isotype control antibody. (F) Genomic snapshots of classical RING1B-bound loci, showing cChIP-seq for RING1B in wild-type cells and H2AK119ub1 in PRC1^CPM^ and PRC1^CKO^ cells. (G) Heatmap analysis of H2AK119ub1 cChIP-seq at RING1B-bound sites in PRC1^CPM^ and PRC1^CKO^ cells. Genomic regions were sorted as in Figure 1E. (H) Metaplot analysis of data shown in (G). See also Figure S2.

Given that we were unable to generate constitutive RING1A^I50A/D53K^;RING1B^I53A/D56K^ ESCs, we set out to develop an ESC line in which removal of PRC1 catalysis could be conditionally induced. To do this, we engineered both endogenous *Ring1b* alleles to contain an I53A/D56K version of the exon encoding the E2 interaction domain in the antisense orientation downstream of the corresponding wild-type exon (Figure 2A). This wild-type/mutant exon pair was flanked by inverted double LoxP/Lox2272 sites, and cells were engineered to express tamoxifen-inducible CRE recombinase. In the absence of tamoxifen (OHT), wild-type RING1B would be expressed, but OHT addition would lead to an inversion event and expression of catalytically inactive RING1B^I53A/D56K^ (Figures 2A and 2B). Importantly, to eliminate the contribution of RING1A, we also constitutively introduced the I50A/D53K mutations into both alleles of endogenous *Ring1a* (Figure 2B).

To validate the functionality of the PRC1 conditional point mutant (PRC1^CPM^) system, we confirmed that *Ring1b* mRNA encoding I53A/D56K was exclusively expressed after 72 h OHT treatment (Figures S2A–S2C). Following OHT treatment, RING1B protein levels were largely unchanged, and RING1B^I53A/D56K^ still occupied Polycomb target sites as assessed by cChIP-seq (Figures 2C and S2D). Furthermore, the levels of other PRC1 subunits were largely unaffected (Figure 2D), and RING1B^I53A/D56K^ was able to form cPRC1 and vPRC1 complexes (Figure 2E). However, in contrast to constitutive RING1B^I53A^ and RING1B^I53A/D56K^ mutant cells, we now observed a complete loss of H2AK119ub1 in the OHT-treated PRC1^CPM^ cells as evident in bulk histone western blot analysis (Figure 2C). We further confirmed a complete loss of H2AK119ub1 genome-wide using cChIP-seq, with H2AK119ub1 no longer found at sites enriched for RING1B or throughout the genome (Figures 2F–2H, S2E, and S2H).

To compare the defects that arise from catalytic inactivation with those that manifest from complete removal of PRC1, we also developed an isogenic ESC line in which both copies of *Ring1a* were constitutively deleted, the first coding exon of both *Ring1b* alleles was flanked by parallel LoxP sites, and OHT-inducible CRE was expressed (Figures 2B and S2F). In this PRC1 conditional knockout (PRC1^CKO^) cell line, RING1B was completely removed following 72 h OHT treatment as evident in western blot and cChIP-seq analysis at Polycomb target sites (Figures 2C and S2D). Importantly, loss of H2AK119ub1 in the OHT-treated PRC1^CKO^ cells was identical to that observed in the PRC1^CPM^ cells (Figures 2C, 2F–2H, S2G, and S2H). However, in contrast to the PRC1^CPM^ cells, cPRC1-specific subunits were almost undetectable when RING1A/B were removed, suggesting that the stability of these proteins requires an intact PRC1 complex (Figure 2D). The combination of isogenic PRC1^CPM^ and PRC1^CKO^ ESC lines (Figure 2B) now provided us with the opportunity to directly test the contribution of PRC1 catalysis to Polycomb chromatin domain formation, and ultimately define whether catalysis by PRC1 is required for gene repression by the Polycomb system.

### PRC2 Binding and Deposition of H3K27me3 at Polycomb Target Sites Is Disrupted in the Absence of PRC1 Catalysis

**Figure 3:**
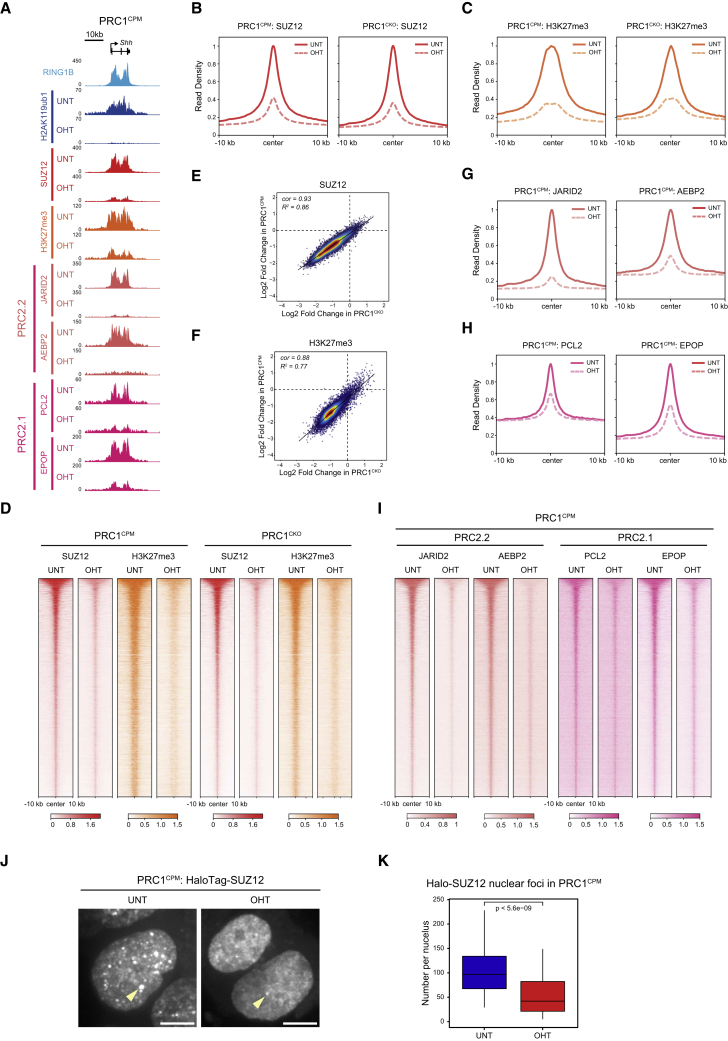
PRC2 Binding and H3K27me3 Deposition at Polycomb Target Sites Rely on PRC1 Catalytic Activity (A) Genomic snapshot of a Polycomb target gene showing cChIP-seq for RING1B, H2AK119ub1, SUZ12, H3K27me3, JARID2, AEBP2, PCL2, and EPOP in PRC1^CPM^ cells. (B) Metaplot analysis of SUZ12 cChIP-seq at PcG-occupied sites in PRC1^CPM^ and PRC1^CKO^ cells. (C) As in (B) for H3K27me3 cChIP-seq. (D) Heatmap analysis of cChIP-seq data shown in (B) and (C). Genomic regions were sorted based on RING1B occupancy in untreated PRC1^CKO^ ESCs. (E) A scatterplot comparing the log2 fold changes in SUZ12 levels at PcG-occupied sites in PRC1^CPM^ and PRC1^CKO^ ESCs. *R*^*2*^ represents coefficient of determination for linear regression and *cor* denotes Pearson correlation coefficient. (F) As in (E) for H3K27me3 cChIP-seq. (G) Metaplot analysis of PRC2.2-specific subunits JARID2 and AEBP2 cChIP-seq at PcG-occupied sites in PRC1^CPM^ cells. (H) As in (G) for PRC2.1-specific subunits PCL2 and EPOP. (I) Heatmap analysis of cChIP-seq data shown in (G) and (H). Genomic regions were sorted as in (D). (J) Maximum intensity projections of JF_549_-Halo-SUZ12 signal in PRC1^CPM^ ESCs. Examples of SUZ12 nuclear foci (Polycomb bodies) are indicated by arrowheads. Scale bar is 5 μm. (K) Boxplots comparing the number of JF_549_-Halo-SUZ12 nuclear foci in PRC1^CPM^ ESCs before (n_cells_ = 55) and after (n_cells_ = 52) OHT treatment. Cells from two independent experiments were analyzed. P values denote statistical significance calculated by a Student’s t test. See also Figure S3.

We have previously demonstrated that PRC1 removal causes a dramatic reduction of PRC2 binding and H3K27me3 at Polycomb target sites, indicating a requirement for PRC1 in normal Polycomb chromatin domain formation (Blackledge et al., 2014, Fursova et al., 2019, Rose et al., 2016). To test whether this requires catalysis, we performed cChIP-seq for SUZ12 and H3K27me3 in the PRC1^CPM^ and PRC1^CKO^ ESCs. In untreated cells, SUZ12 binding occurred at sites also occupied by RING1B (Figures 3A, S3A, and S3B), but strikingly in the OHT-treated PRC1^CPM^ cells we observed a dramatic reduction in the levels of both SUZ12 and H3K27me3 at these sites (Figures 3A–3D, S3C, and S3D). Importantly, this recapitulated the changes observed in the OHT-treated PRC1^CKO^ cells (Figures 3B, 3C, 3E, 3F, S3C, and S3D).

Some residual SUZ12 binding and H3K27me3 remained at target sites in both the OHT-treated PRC1^CKO^ and PRC1^CPM^ cells (Figures 3A–3D). To examine whether this corresponded to retention of either PRC2.1 or PRC2.2, we performed cChIP-seq for JARID2, a PRC2.2-specific subunit that directly binds H2AK119ub1, in the PRC1^CPM^ cells (Cooper et al., 2016, Kalb et al., 2014). Following removal of PRC1 catalysis, we observed a near-complete loss of JARID2 binding at Polycomb target sites (Figures 3A, 3G, 3I, and S3E). We also observed a reduction in JARID2 protein levels in the OHT-treated PRC1^CPM^ cells (Figure S3G), suggesting that chromatin binding may be required for JARID2 stability. Binding of AEBP2, another PRC2.2 subunit, was also lost, confirming that PRC2.2 occupancy requires H2AK119ub1 (Figures 3A, 3G, 3I, and S3E). Next, we examined occupancy of the PRC2.1-specific subunits PCL2 and EPOP following PRC1 catalytic inactivation. This revealed that PCL2 and EPOP binding at Polycomb target sites was reduced in the OHT-treated PRC1^CPM^ cells, albeit to a lesser extent than that of JARID2 and AEBP2 (Figures 3A, 3H, 3I, S3F, and S3H). Importantly, some PRC2.1 occupancy was clearly retained in the absence of PRC1 catalysis, possibly due to the DNA binding activity of PCL1/2/3 proteins (Li et al., 2017, Perino et al., 2018), which contribute to PRC2.1 occupancy at target sites in ESCs (Healy et al., 2019, Højfeldt et al., 2019, Perino et al., 2019).

In the nucleus, PcG proteins are enriched at cytological foci, called Polycomb bodies, which contain Polycomb-repressed genes (Cheutin and Cavalli, 2012, Isono et al., 2013, Saurin et al., 1998). To examine whether the major reductions in PRC2 enrichment observed at target sites in fixed cells by cChIP-seq were also evident in live cells, we examined nuclear localization of endogenous SUZ12 protein fused with HaloTag in PRC1^CPM^ cells. In untreated PRC1^CPM^ cells, we observed approximately one hundred SUZ12 nuclear foci per cell with a wide range of sizes and intensities (Figures 3J, 3K, and S3I). Following loss of PRC1 catalytic activity, the number, size, and intensity of SUZ12 foci were dramatically reduced, indicating that normal PRC2 localization in live cells was disrupted (Figures 3J, 3K, S3I, and S3J).

### PRC1 Catalytic Activity Is Required for Canonical PRC1 Occupancy and Long-Range Polycomb Chromatin Domain Interactions

**Figure 4:**
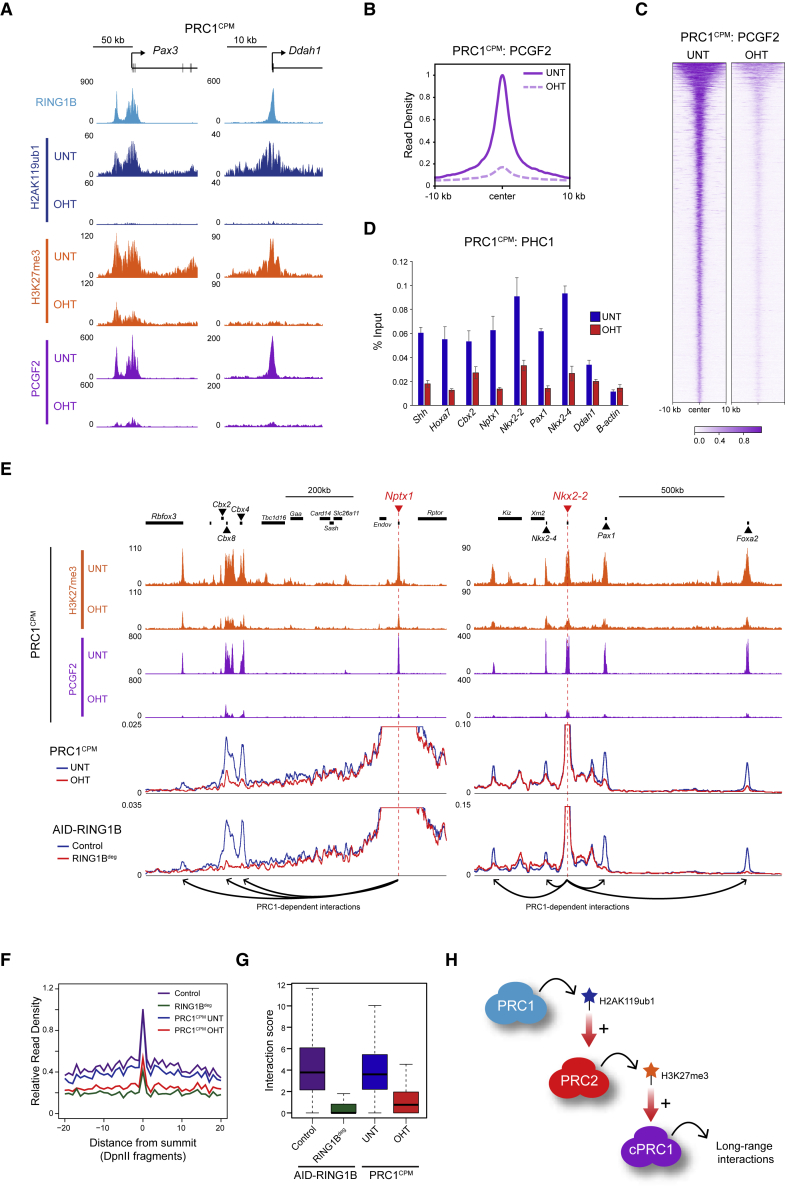
PRC1 Catalytic Activity Drives Canonical PRC1 Occupancy and Long-Range Polycomb Chromatin Domain Interactions (A) Genomic snapshots of Polycomb target genes, showing cChIP-seq for RING1B, H2AK119ub1, H3K27me3, and PCGF2 (cPRC1) in PRC1^CPM^ cells. (B) Metaplot analysis of PCGF2 cChIP-seq at PCGF2 target sites in PRC1^CPM^ cells. (C) Heatmap analysis of data shown in (B). Genomic regions were sorted based on RING1B occupancy in untreated PRC1^CKO^ ESCs. (D) ChIP-qPCR for PHC1 at a panel of Polycomb target genes in PRC1^CPM^ cells. B-actin is an active gene not bound by PHC1. Error bars show SEM (n = 3). (E) Genomic snapshots of two regions encompassing classical Polycomb target sites used as baits in CaptureC (bait probe positions are marked by dashed red lines). H3K27me3 and PCGF2 cChIP-seq data in PRC1^CPM^ cells are shown at the top. Below is the bait interaction landscape measured by CaptureC in PRC1^CPM^ cells (untreated and OHT-treated) and auxin-treated *Ring1a*^*−/−*^;AID-RING1B (RING1B^deg^) ESCs (relative to the Control cell line with intact PRC1). Arrows illustrate long-range PRC1-dependent interactions. (F) Meta-analysis of interactions between bait Polycomb target regions and other PCGF2 target sites (n = 130) in cells described in (E). Read density is shown relative to the untreated PRC1^CPM^ cells. (G) Boxplot analysis of CHiCAGO scores for the interactions between the bait Polycomb target regions and other PCGF2 target sites (n = 130) for cells described in (E). (H) A schematic summarizing the model in which deposition of H2AK119ub1 by PRC1 drives accumulation of PRC2 and deposition of H3K27me3 at target sites, promoting cPRC1 binding and long-range interactions between these regions. See also Figure S4.

At Polycomb target sites, PRC2-deposited H3K27me3 is recognized by cPRC1 complexes, which can mediate long-range interactions between Polycomb chromatin domains. Previous work has reported that these interactions are diminished in the absence of RING1B but intact in RING1B^I53A^ cells (Eskeland et al., 2010, Kundu et al., 2017), leading to the conclusion that Polycomb chromatin domain interactions do not require PRC1 catalysis. Given the hypomorphic nature of RING1B^I53A^ and our observation that H3K27me3 levels are dramatically reduced in the OHT-treated PRC1^CPM^ cells, we wanted to determine whether cPRC1 binding and long-range Polycomb chromatin domain interactions were affected when PRC1 catalysis was completely removed. To this end, we carried out cChIP-seq for PCGF2, a core component of cPRC1 complexes in ESCs (Morey et al., 2015). In untreated PRC1^CPM^ cells, PCGF2 was enriched at RING1B-bound regions with high levels of PRC2 and H3K27me3 (Figures 4A, S4A, and S4B). Importantly, following OHT treatment, PCGF2 occupancy at target sites was majorly reduced, as was the binding of the cPRC1 component PHC1 (Figures 4A–4D and S4C).

Next, we used CaptureC (Hughes et al., 2014) to examine interaction profiles for 24 classical Polycomb target genes that are highly enriched with PCGF2 and RING1B (Figure S4D). Strikingly, this revealed that long-range interactions between these regions and other classical Polycomb target sites were largely abolished in the OHT-treated PRC1^CPM^ cells, and this effect was highly comparable to that observed following complete removal of PRC1 using a degron approach (Figures 4E–4G and S4E) (Rhodes et al., 2019). Together, these observations support a model whereby PRC1 catalytic activity drives a sequence of downstream events, including PRC2 binding, deposition of H3K27me3, and recruitment of canonical PRC1, which culminate in the formation of Polycomb chromatin domains that can engage in long-range interactions (Figure 4H).

### Variant PRC1 Complexes Occupy Polycomb Target Sites Independently of PRC1 Catalysis

**Figure 5:**
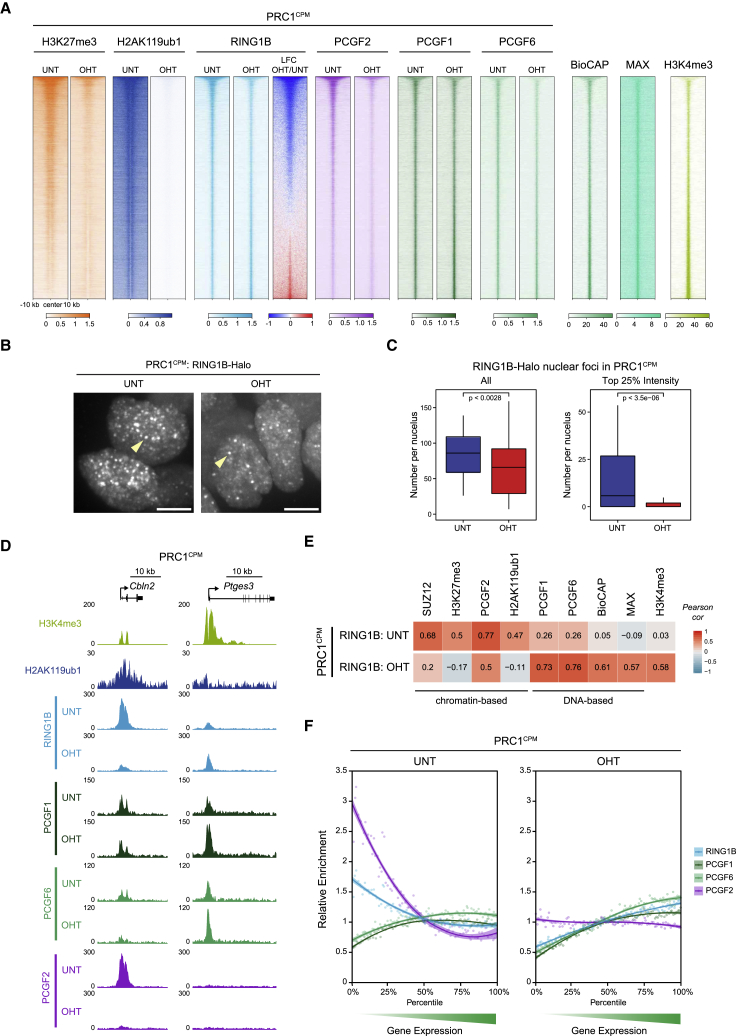
Variant PRC1 Complexes Occupy Polycomb Target Sites Independently of PRC1 Catalysis (A) Heatmap analysis of cChIP-seq for H3K27me3, H2AK119ub1, RING1B, PCGF2, PCGF1, and PCGF6 at RING1B-bound sites in PRC1^CPM^ cells. Also shown is BioCAP-seq (measure of non-methylated CpG-rich DNA) and ChIP-seq for MAX and H3K4me3 in wild-type ESCs. Genomic intervals were sorted based on log2 fold change in RING1B occupancy following OHT treatment in PRC1^CPM^ ESCs. (B) Maximum intensity projections of RING1B-Halo-JF_549_ signal in PRC1^CPM^ ESCs. Examples of RING1B nuclear foci are indicated by arrowheads. Scale bar is 5 μm. (C) Boxplots comparing the number of all (left panel) or top 25% highest intensity (right panel) RING1B-Halo-JF_549_ foci per nucleus in PRC1^CPM^ ESCs before (n_cells_ = 69) and after (n_cells_ = 83) OHT treatment. Cells from two independent experiments were analyzed. P values denote statistical significance calculated by a Student’s t test. (D) Genomic snapshots of two genes showing cChIP-seq for RING1B, PCGF1, PCGF6, and PCGF2 in PRC1^CPM^ ESCs. H2AK119ub1 cChIP-seq in untreated cells and H3K4me3 ChIP-seq in wild-type ESCs is also shown. *Cbln2* is a lowly transcribed gene with high-level RING1B occupancy and *Ptges3* is a more highly transcribed gene. (E) Correlation of cChIP-seq signal for RING1B with PRC2 (SUZ12 and H3K27me3) and PRC1 (PCGF2, PCGF1, PCGF6, and H2AK119ub1) in untreated and OHT-treated PRC1^CPM^ ESCs. Correlation with BioCAP-seq and ChIP-seq for MAX and H3K4me3 in wild-type ESCs is also shown. (F) Relative enrichment of RING1B, PCGF1, PCGF6, and PCGF2 cChIP-seq signal in PRC1^CPM^ ESCs across promoter-proximal RING1B-bound sites divided into percentiles based on the expression level of the associated gene in untreated PRC1^CPM^ cells. For each factor, enrichment is shown relative to the fiftieth percentile. Lines represent smoothed conditional means based on loess local regression fitting. See also Figure S5.

While cPRC1 complexes account for a significant proportion of RING1B occupancy at established Polycomb chromatin domains (Fursova et al., 2019, Morey et al., 2015), this is a downstream consequence of PRC1 catalysis (Figure 4). Therefore, we reasoned that in the absence of PRC1 catalytic activity and cPRC1 binding, the mechanisms responsible for PRC1 target site selection may be unmasked. To examine this possibility, we examined RING1B occupancy in PRC1^CPM^ cells before and after OHT treatment and compared this to the binding of other Polycomb factors and chromatin features. In untreated PRC1^CPM^ cells, RING1B binding correlated strongly with levels of PCGF2 and PRC2, consistent with a prominent role for cPRC1 in shaping RING1B occupancy (Figures 5A, 5D, 5E, S5A, S5E, and S5F). Following OHT treatment, RING1B binding was majorly reduced at sites that normally have high levels of PRC1 and PRC2 and now correlated only modestly with cPRC1 or PRC2 occupancy (Figures 5A, 5D, 5E, S5A, S5B, S5E, and S5F). Interestingly, RING1B levels were unchanged or even increased in OHT-treated PRC1^CPM^ cells at a large number of sites that normally have low to moderate enrichment of PRC1 and PRC2 (Figures 5A, 5D, S5A, and S5B). Similar effects were observed by live-cell imaging of Polycomb foci in untreated and OHT-treated PRC1^CPM^ cells in which we added a HaloTag to endogenous RING1B (Figures 5B and S5D). Following loss of PRC1 catalysis, the total number, average size, and intensity of nuclear RING1B foci were only modestly reduced (Figures 5B, 5C, and S5C). However, we observed a dramatic reduction in the number of bright RING1B foci in OHT-treated PRC1^CPM^ cells, while the number of less intense foci was largely unaffected (Figures 5B, 5C, S5C, and S5D). Together, this indicates that while high-level enrichment of RING1B at Polycomb chromatin domains requires PRC1 catalytic activity, there exists low-level RING1B binding across all target sites that is independent of PRC1 catalysis.

To explore the features associated with RING1B occupancy in the absence of PRC1 catalytic activity, we focused on the PCGF1- and PCGF6-containing vPRC1 complexes, both of which have DNA-binding modules. PCGF1-PRC1 contains KDM2B, a ZF-CXXC domain protein that binds non-methylated CpGs (Farcas et al., 2012, He et al., 2013, Wu et al., 2013), while PCGF6-PRC1 incorporates MAX/MGA DNA-binding factors (Gao et al., 2012, Ogawa et al., 2002). In untreated PRC1^CPM^ cells, cChIP-seq for PCGF1 and PCGF6 revealed a broad and uniform binding of both factors across all RING1B-occupied sites, which correlated poorly with RING1B levels (Figures 5A, 5D, 5E, S5A, and S5G). Importantly, in the OHT-treated PRC1^CPM^ cells, we observed a strong correlation between RING1B occupancy and binding of PCGF1 and PCGF6, as well as with non-methylated CpG-rich DNA and MAX binding (Figures 5A, 5E, S5G, and S5H). Furthermore, occupancy of PCGF1 and PCGF6 was only modestly affected by loss of PRC1 catalytic activity, indicating that binding of these vPRC1 complexes is largely independent of PRC1 catalysis and Polycomb chromatin domain formation (Figures 5A, 5D, S5A, and S5B).

We and others have proposed that transcription may counteract formation of Polycomb chromatin domains (Jermann et al., 2014, Klose et al., 2013, Riising et al., 2014, Vernimmen et al., 2011). In support of this idea, in untreated PRC1^CPM^ cells, the highest levels of RING1B and PCGF2 were associated with promoters of lowly transcribed genes that were enriched for H3K27me3 and H2AK119ub1, but not H3K4me3 (Figures 5A, 5F, S5I, and S5J). In contrast, more actively transcribed RING1B targets exhibited low levels of H2AK119ub1, PRC2, H3K27me3, and cPRC1, despite being occupied by similar levels of the PCGF1- and PCGF6-containing vPRC1 complexes. Importantly, in OHT-treated PRC1^CPM^ cells, high RING1B enrichment at lowly transcribed genes was lost, uncovering a uniform RING1B distribution across target sites that mirrored PCGF1 and PCGF6 occupancy (Figures 5A, 5D, 5F, S5I, and S5J). Together, these observations are consistent with a model in which the PCGF1- and PCGF6-containing vPRC1 complexes broadly engage with target sites via their DNA-binding domains, which, in the absence of counteracting features associated with active transcription, leads to high-level H2AK119ub1 deposition and Polycomb chromatin domain formation.

### The Catalytic Activity of PRC1 Is Required for Polycomb-Mediated Gene Repression

**Figure 6:**
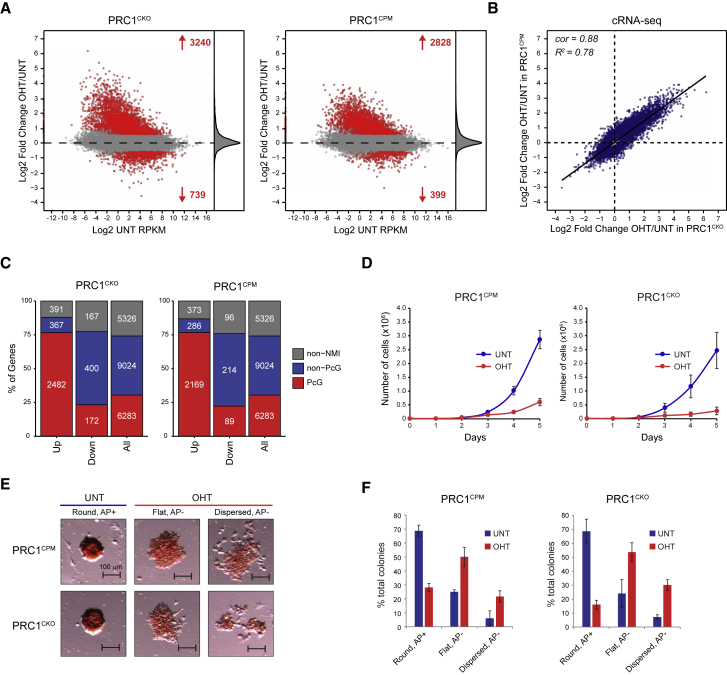
PRC1 Catalytic Activity Is Required for PRC1-Mediated Gene Repression and ESC Viability (A) MA-plots of log2 fold changes in gene expression (cRNA-seq) in PRC1^CKO^ and PRC1^CPM^ ESCs. Significant gene expression changes (p-adj < 0.05 and > 1.5-fold) are shown in red. Density of gene expression changes is shown on the right. (B) A scatterplot comparing the log2 fold changes in gene expression (cRNA-seq) in PRC1^CKO^ and PRC1^CPM^ ESCs for all genes. *R*^*2*^ represents coefficient of determination for linear regression and *cor* denotes Pearson correlation coefficient. (C) A bar plot illustrating the distribution of gene expression changes (p-adj < 0.05 and > 1.5-fold) in PRC1^CKO^ and PRC1^CPM^ ESCs between different gene classes: genes lacking a non-methylated CGI (Non-NMI), non-Polycomb-occupied genes (Non-PcG), and Polycomb-occupied genes (PcG). (D) A growth curve assay in PRC1^CPM^ and PRC1^CKO^ ESCs. Error bars show SEM (n = 6). (E) Examples of typical ESC colony morphology and alkaline phosphatase (AP) staining in PRC1^CPM^ and PRC1^CKO^ ESCs. Untreated colonies for both cell lines were typically round with high levels of AP activity (Round, AP+), while following OHT treatment colonies were either flat with lower levels of AP activity (Flat, AP−) or dispersed with lower levels of AP activity (Dispersed, AP−). (F) Quantification of morphology and AP staining in PRC1^CPM^ and PRC1^CKO^ ESCs according to classification in (E). For each cell line (UNT and OHT), at least 100 colonies were counted. Error bars show SEM (n = 3). See also Figure S6.

To address whether catalysis by PRC1 is required for transcriptional repression, we carried out calibrated RNA-seq (cRNA-seq) in the PRC1^CPM^ and PRC1^CKO^ ESCs before and after OHT treatment. Loss of PRC1 in PRC1^CKO^ cells resulted in derepression of approximately 3,000 genes, most of which were Polycomb target genes (Figures 6A and 6C). This is consistent with PRC1 playing the central role in Polycomb-mediated gene repression in ESCs (Endoh et al., 2008, Fursova et al., 2019). Strikingly, removal of PRC1 catalysis resulted in derepression of a similar number of genes, and a comparable proportion of these were Polycomb targets (Figures 6A and 6C). A more detailed comparison of the gene expression alterations following OHT treatment in PRC1^CKO^ and PRC1^CPM^ ESCs revealed a strong correlation in the magnitude of gene expression changes between these cell lines, both for all genes and for PRC1-repressed genes (Figures 6B, S6A, and S6B). Together, these observations indicate that PRC1 catalytic activity is central to Polycomb-mediated gene repression.

In agreement with PRC1 being essential for mouse ESC viability (Endoh et al., 2008), OHT-treated PRC1^CKO^ cells exhibited a marked reduction in proliferation (Figure 6D). Importantly, this effect was largely recapitulated in the OHT-treated PRC1^CPM^ cells, highlighting an essential role for PRC1 catalytic activity in ESC viability. Furthermore, following removal of PRC1 catalysis, we observed a change in ESC morphology and a reduction in alkaline phosphatase (AP) staining (Figures 6E and 6F). This effect was comparable to that observed in the OHT-treated PRC1^CKO^ cells, albeit slightly lesser in magnitude (Figures 6E and 6F). However, importantly, both in the OHT-treated PRC1^CPM^ and PRC1^CKO^ cells, the expression of key pluripotency factors was only modestly affected (Figure S6C), implying only a partial exit from pluripotency. This suggests that the majority of gene expression changes observed during the short time frame of our conditional perturbations are a direct consequence of PRC1 removal or PRC1 catalytic inactivation and not a secondary effect of ESC differentiation. This is further supported by the fact that approximately 75% of the reactivated genes in the OHT-treated PRC1^CKO^ and PRC1^CPM^ cells corresponded to classical Polycomb target genes occupied by both RING1B and SUZ12 (Figure 6C).

**Figure 7:**
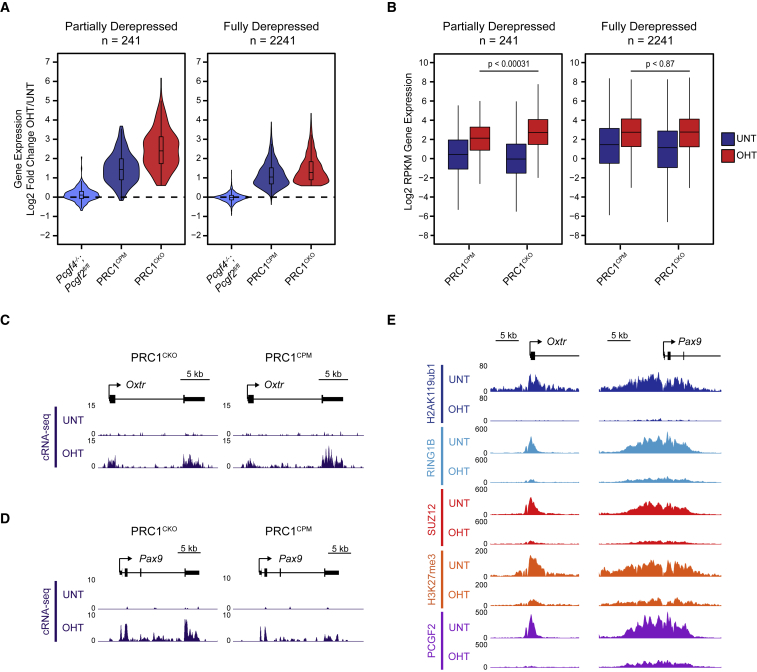
A Small Subset of PRC1 Target Genes Are Not Fully Derepressed Following Loss of PRC1 Catalysis (A) A violin plot comparing log2 fold changes in expression (cRNA-seq) of PRC1-repressed genes that are partially (n = 241) or fully derepressed (n = 2,241) in PRC1^CPM^ as compared to PRC1^CKO^ ESCs following OHT treatment in *Pcgf4*^*−/−*^*;Pcgf2*^*fl/fl*^, PRC1^CPM^, and PRC1^CKO^ ESC lines. (B) A boxplot of expression levels (cRNA-seq) of genes described in (A) in PRC1^CPM^ and PRC1^CKO^ ESCs. P values denote statistical significance calculated by a Wilcoxon rank sum test. (C) A genomic snapshot of a PRC1-repressed gene that is fully derepressed in OHT-treated PRC1^CPM^ as compared to PRC1^CKO^ ESCs, showing gene expression (cRNA-seq) in PRC1^CKO^ and PRC1^CPM^ ESCs. (D) As in (C) for a PRC1-repressed gene that is partially derepressed in OHT-treated PRC1^CPM^ as compared to PRC1^CKO^ ESCs. (E) Genomic snapshots for genes described in (C) and (D) showing cChIP-seq for PRC1 (RING1B, H2AK119ub1, and PCGF2) and PRC2 (SUZ12 and H3K27me3) in PRC1^CPM^ ESCs. See also Figure S7.

Loss of PRC1 catalytic activity largely recapitulated the gene expression defects that manifest when PRC1 is completely removed (Figures 6B, S6A, and S6B). However, we found a small number of PRC1-repressed genes (241) which were derepressed to a lesser extent in the OHT-treated PRC1^CPM^ compared to the PRC1^CKO^ ESCs (Figures 7A, 7B, 7D, and 7E). These tended to have large Polycomb chromatin domains that were highly enriched with cPRC1 and H3K27me3 in untreated cells and retained low levels of cPRC1 binding in the OHT-treated PRC1^CPM^ cells (Figures 7E and S7A–S7C). This suggests that residual cPRC1 present at the promoters of these genes may contribute to their silencing in the absence of PRC1 catalysis. Nevertheless, repression of this small subset of genes still heavily relied on PRC1 catalytic activity, as removal of cPRC1 alone caused only a modest increase in their expression (Figure 7A). Importantly, the remaining PRC1-repressed genes were reactivated to the same level in the OHT-treated PRC1^CPM^ and PRC1^CKO^ ESCs (Figures 7B and 7C), indicating that PRC1 catalysis is a central determinant of Polycomb-mediated gene repression. Therefore, through systematically dissecting the requirement for PRC1 catalytic activity in Polycomb system function, we provide direct evidence that this activity is essential for gene repression.

## Discussion

Understanding the extent to which catalysis by chromatin-modifying enzymes contributes to their function is an ongoing challenge in the field of chromatin biology. Addressing this question requires *in vitro* biochemical characterization of inactivating mutations to show that they cause complete loss of catalysis without protein complex disruption. Furthermore, this must be combined with quantitative measurements of the modified substrate *in vivo* to ensure that the product of catalysis is completely lost in the cellular context. Here, we satisfy these two central requirements in studying PRC1 catalytic activity, whose contribution to Polycomb system function has remained controversial. Importantly, we discover that catalysis by PRC1 is essential for gene repression and normal Polycomb chromatin domain formation. While we have limited our investigation to ESCs, a recent study has also examined the contribution of PRC1 catalytic activity to gene regulation in a neuronal cell fate restriction model (Tsuboi et al., 2018). Through expression analysis of a handful of Polycomb target genes, this previous study reported that PRC1 catalysis is required for gene repression during early neurogenesis, while catalysis-independent gene repression predominated at later astrogliogenic stages. This suggests that catalysis-independent mechanisms may contribute to the maintenance or fidelity of gene repression as cell linage commitment proceeds. Therefore, moving forward, it will be important to use genome-wide approaches to examine how catalysis by PRC1 contributes to Polycomb system function in more differentiated cell types and during early development. Furthermore, the effects of fully inactivating catalytic mutations should also be examined in other model organisms, such as *Drosophila,* where the contribution of PRC1 catalysis to Polycomb target gene repression and embryonic development has been proposed to be more limited (Pengelly et al., 2015).

Defining the mechanisms of Polycomb target site selection and Polycomb chromatin domain formation is central to understanding the logic by which the Polycomb system functions. In mammalian cells, high-level Polycomb occupancy occurs at CGI elements (Mikkelsen et al., 2007), and ectopic CpG-rich DNA is sufficient to establish new Polycomb chromatin domains *de novo* (Jermann et al., 2014, Lynch et al., 2012, Mendenhall et al., 2010). A mechanistic link between CGIs and Polycomb recruitment came with the discovery that KDM2B, a component of the PCGF1-containing vPRC1 complex, has DNA-binding activity that is specific for non-methylated CpG dinucleotides, suggesting that recognition of CGI DNA may underpin Polycomb target site selection (Farcas et al., 2012, He et al., 2013, Wu et al., 2013). Similarly, the PCGF6-containing vPRC1 complex has DNA-binding activities that can contribute to Polycomb occupancy at CGIs (Endoh et al., 2017, Scelfo et al., 2019, Stielow et al., 2018). Furthermore, it has also recently been reported that PRC2.1-specific PCL subunits have DNA-binding activity specific for CGIs (Li et al., 2017, Perino et al., 2018). While PCGF1 and PCGF6 broadly occupy CGI-associated target sites, somewhat paradoxically, only a subset of these sites achieve high levels of PRC1, H2AK119ub1, PRC2, and H3K27me3. Here, we provide evidence that PCGF1- and PCGF6-containing vPRC1 complexes engage with or sample potential target sites independently of PRC1 catalytic activity. We propose that high-level deposition of H2AK119ub1 only occurs at a receptive subset of genes with low transcriptional activity, where it supports formation of Polycomb chromatin domains at which PRC1 and PRC2 mutually reinforce each other’s occupancy. These ideas are consistent with previous work showing that the Polycomb system responds to, rather than instructs, the transcriptional state of a gene (Berrozpe et al., 2017, Hosogane et al., 2013, Riising et al., 2014, Vernimmen et al., 2011). Through this target site selection logic, the mammalian Polycomb complexes would converge on lowly transcribed genes where they could function to protect against stochastic gene activation events that may otherwise be deleterious to the maintenance of cell-type-specific transcriptional programs (Klose et al., 2013).

The responsive nature of the Polycomb system raises the question of whether some of the effects on Polycomb chromatin domain formation that we observe in the absence of PRC1 catalysis may manifest from transcription-associated eviction of Polycomb complexes (Beltran et al., 2019). For example, do the major reductions in PRC2 occupancy and H3K27me3 at Polycomb target sites directly result from loss of PRC1 catalysis and a breakdown of PRC2-dependent recognition of H2AK119ub1, or are they simply a consequence of gene activation? While it is difficult to distinguish between these two possibilities at genes that change in expression, following removal of PRC1 catalysis, a proportion of genes showed reduced PRC2 binding and H3K27me3 in the absence of gene expression changes. Importantly, the reductions in PRC2 and H3K27me3 at these genes were very similar to the reductions observed at genes that increased in expression (Figures S7D and S7E). This suggests that PRC1 catalytic activity directly supports PRC2 occupancy and H3K27me3 at Polycomb chromatin domains, consistent with ectopic tethering experiments (Blackledge et al., 2014).

Our observations reveal that PRC1 catalysis is essential for Polycomb-mediated gene repression, but how is this achieved mechanistically? We recently demonstrated that removal of cPRC1 complexes in ESCs has little effect on gene repression (Fursova et al., 2019), while earlier work showed that PRC2 removal resulted in few gene expression defects (Riising et al., 2014). This suggests that the mechanisms by which PRC1 catalysis represses transcription do not rely solely on PRC2 and cPRC1 in ESCs, despite the fact that these complexes are clearly involved in gene repression in some other contexts (Akasaka et al., 2001, Moussa et al., 2019, O’Carroll et al., 2001, Pasini et al., 2007). One possibility is that H2AK119ub1 directly disrupts RNA Pol II activity (Stock et al., 2007, Zhou et al., 2008), as suggested by *in vitro* transcription assays (Aihara et al., 2016, Nakagawa et al., 2008). Alternatively, H2AK119ub1 could recruit reader proteins that elicit gene repression (Ali et al., 2018, Cooper et al., 2016, Qin et al., 2015, Richly et al., 2010, Zhang et al., 2017) or interfere with deposition of histone modifications that facilitate transcription (Nakagawa et al., 2008, Yuan et al., 2013). Finally, it is possible that PRC1 drives gene repression via ubiquitylation of histone H2A variants (Surface et al., 2016) or other non-histone substrates (Ben-Saadon et al., 2006). Future work focused on defining the mechanisms by which the catalytic activity of PRC1 counteracts RNA Pol II activity and gene expression will be important. Nevertheless, our new discoveries place PRC1 catalysis at the forefront of Polycomb-mediated gene repression in ESCs, paving the way for more detailed mechanistic understanding of Polycomb system function.

## STAR★Methods

### Key Resources Table

**Table:** 

REAGENT or RESOURCE	SOURCE	IDENTIFIER
**Antibodies**		

Rabbit monoclonal anti-H2AK119ub1	Cell Signaling Technology	Cat# 8240; RRID:AB_10891618
Rabbit polyclonal anti-H3K27me3	In house (Rose et al., 2016)	n/a
Rabbit polyclonal anti-H3K4me3	In house (Farcas et al., 2012)	n/a
Rabbit polyclonal anti-H2A	Millipore	Cat# 07-146; RRID:AB_11212920
Mouse monoclonal anti-H3	Cell Signaling Technology	Cat# 3638; RRID:AB_1642229
Mouse monoclonal anti-H4	Cell Signaling Technology	Cat# 2935; RRID:AB_1147658
Rabbit monoclonal anti-RING1B (ChIP)	Cell Signaling Technology	Cat# 5694; RRID:AB_20705604
Mouse monoclonal anti-RING1B (WB)	Atsuta et al., 2001	n/a
Rabbit monoclonal anti-SUZ12	Cell Signaling Technology	Cat# 3737; RRID:AB_2196850
Rabbit monoclonal anti-EZH2	Cell Signaling Technology	Cat# 5246; RRID:AB_10694683
Rabbit monoclonal anti-JARID2	Cell Signaling Technology	Cat# 13594; RRID:AB_2798269
Rabbit polyclonal anti-PCL2 (M96)	GenWay	Cat# GWB-FA7207; RRID:AB_10511542
Rabbit polyclonal anti-EPOP (C17orf96)	Active motif	Cat# 61753; RRID:AB_2793758
Rabbit monoclonal anti-AEBP2	Cell Signaling Technology	Cat# 14129; RRID:AB_2798398
Rabbit polyclonal anti-PCGF6	LifeSpan BioSciences	Cat# LS-C482495
Rabbit polyclonal anti-PCGF2 (Mel-18)	Santa Cruz	Cat# sc-10744; RRID:AB_2267885
Rabbit polyclonal anti-PCGF1	In house (Blackledge et al., 2014)	n/a
Mouse monoclonal anti-PHC1	Cell Signaling Technology	Cat# 13768; RRID:AB_2716803
Rabbit polyclonal anti-CBX7 (WB)	Millipore	Cat# 07-981; RRID:AB_10807034
Rabbit polyclonal anti-KDM2B	In house (Farcas et al., 2012)	n/a
Rabbit polyclonal anti-RYBP (DEDAF)	Millipore	Cat# AB3637; RRID:AB_2285466
Rabbit monoclonal anti-BRG1	Abcam	Cat# ab110641; RRID:AB_10861578

**Chemicals, Peptides, and Recombinant Proteins**		

Methanol-free Formaldehyde	Thermo Fisher Scientific	Cat# 10751395
DSG	Thermo Fisher Scientific	Cat# 11836794
Micrococcal Nuclease	Thermo Fisher Scientific	Cat# EN0181
Proteinase K	Sigma	Cat# P4850
(Z)-4-Hydroxytamoxifen	Sigma	Cat# H7904
SensiMix SYBR No-ROX Kit	Bioline	Cat# QT650-20
Lipofectamine 3000	Thermo Fisher Scientific	Cat# L3000015
Indole-3-acetic acid sodium salt	Sigma	Cat# I5148
Fluorobrite DMEM	Thermo Fisher Scientific	Cat# A1896701
UBE1	Boston Biochem	Cat# E-305
UbcH5c	Enzo Life Sciences	Cat# BML-UW9070
Methylated ubiquitin	Boston Biochem	Cat# U-501
500 nm Janelia Fluor 549 (JF_549_)	Grimm et al., 2017	n/a
Hoechst 33258	Thermo fisher Scientific	Cat# H3569

**Critical Commercial Assays**		

NEBNext® Multiplex Oligos for Illumina® (Index Primers Set 1)	NEB	Cat# E7335L
NEBNext® Multiplex Oligos for Illumina® (Index Primers Set 2)	NEB	Cat# E7500L
NEBNext® Ultra™ DNA Library Prep Kit for Illumina®	NEB	Cat# E7370L
NEBNext® Ultr™ II FS DNA Library Prep Kit for Illumina	NEB	Cat# E7805L
NEBNext® Ultra™ II Directional RNA Library Prep Kit for Illumina®	NEB	Cat# E7760L
NEBNext rRNA Depletion Kit (HumanMouseRat)	NEB	Cat# E6310L
High Sensitivity DNA Kit for Bioanalyzer	Agilent	Cat# 5067-4626
RNA Pico 6000 Kit for Bioanalyzer	Agilent	Cat# 5067-1513
TURBO DNA-*free*™ Kit	Thermo Fisher Scientific	Cat# AM1907
Gibson Assembly Master Mix	NEB	Cat# E2611L
NextSeq® 500/550 High Output Kit v2 (150 cycles)	Illumina	Cat# FC-404-2002
NextSeq 500/550 High Output v2 Kit (75 cycles)	Illumina	Cat# FC-404-2005
RNeasy Mini Kit	QIAGEN	Cat# 74106
Quick-DNA Miniprep Kit	Zymo Research	Cat# D3024
KAPA Illumina DNA Standards	Roche	Cat# 7960387001
ChIP DNA Clean and Concentrator	Zymo Research	Cat# D5205

**Deposited Data**		

GEO: GSE132754	SuperSeries	GEO
GEO: GSE132752	cChIP-seq	GEO
GEO: GSE132753	cRNA-seq	GEO
GEO: GSE132751	CaptureC	GEO

**Experimental Models: Cell Lines**		

Mouse ESC: PRC1^CPM^	This study	n/a
Mouse ESC: PRC1^CKO^	This study	n/a
Mouse ESC: *Pcgf4*^*−/−*^*;Pcgf2*^*fl/fl*^	Fursova et al., 2019	n/a
Mouse ESC: HaloTag-SUZ12;PRC1^CPM^	This study	n/a
Mouse ESC: RING1B-HaloTag;PRC1^CPM^	This study	n/a
Mouse ESC: *Ring1a*^*−/−*^;AID-RING1B (RING1B^deg^)	Rhodes et al., 2019	n/a
Mouse ESC: Control TIR1-only	Rhodes et al., 2019	n/a

**Software and Algorithms**		

SAMtools (v1.7)	Li et al., 2009	http://www.htslib.org/
BEDtools (v2.17.0)	Quinlan, 2014	http://bedtools.readthedocs.io/en/latest/
Bowtie 2 (v2.3.4)	Langmead and Salzberg, 2012	http://bowtie-bio.sourceforge.net/bowtie2/index.shtml
Sambamba (v0.6.7)	Tarasov et al., 2015	http://lomereiter.github.io/sambamba/
STAR (v2.5.4)	Dobin et al., 2013	https://github.com/alexdobin/STAR
deepTools (v3.1.1)	Ramírez et al., 2014	https://deeptools.readthedocs.io/en/develop/
MACS2 (v2.1.1)	Zhang et al., 2008	https://github.com/taoliu/MACS/tree/master/MACS2
UCSC Genome Browser	Kent et al., 2002	https://genome.ucsc.edu/
Bioconductor (v3.6)	Huber et al., 2015	https://www.bioconductor.org/
DESeq2	Love et al., 2014	https://bioconductor.org/packages/release/bioc/html/DESeq2.html
HOMER	Heinz et al., 2010	http://homer.ucsd.edu/homer/
HiCUP	Wingett et al., 2015	https://www.bioinformatics.babraham.ac.uk/projects/hicup/
CHiCAGO	Cairns et al., 2016	http://regulatorygenomicsgroup.org/chicago/
TANGO	Ollion et al., 2013	https://biophysique.mnhn.fr/tango/

### Lead Contact and Materials Availability

Further information and requests for resources and reagents should be directed to and will be fulfilled by the Lead Contact, Rob Klose (rob.klose@bioch.ox.ac.uk).

### Experimental Model and Subject Details

Male mouse embryonic stem cells were grown on gelatin-coated plates at 37°C and 5% CO2, in Dulbecco’s Modified Eagle Medium (DMEM) supplemented with 15% fetal bovine serum (Labtech), 2 mM L-glutamine (Life Technologies), 1x penicillin-streptomycin solution (Life Technologies), 1x non-essential amino acids (Life Technologies), 0.5 mM beta-mercaptoethanol (Life Technologies), and 10 ng/mL leukemia inhibitory factor. To induce conditional catalytic point mutation or knockout, PRC1^CPM^ or PRC1^CKO^ cells were treated with 800 nM 4-hydroxytamoxifen (OHT) for 72 h. Cells were regularly tested for the presence of mycoplasma.

*Drosophila* S2 (SG4) cells were grown adhesively at 25°C in Schneider’s *Drosophila* Medium (Life Technologies), supplemented with 1x penicillin-streptomycin solution (Life Technologies) and 10% heat-inactivated fetal bovine serum (Labtech).

Human HEK293T cells were grown at 37°C and 5% CO2, in Dulbecco’s Modified Eagle Medium (DMEM) supplemented with 10% fetal bovine serum (Labtech), 2 mM L-glutamine (Life Technologies), 1x penicillin-streptomycin solution (Life Technologies), and 0.5 mM beta-mercaptoethanol (Life Technologies).

### Method Details

#### Recombinant protein expression and purification

Mouse RING1B, tagged with StrepII and 6xHis tags, was coexpressed with PCGF1 and RYBP from a pST44 polycistronic plasmid in *E. coli* BL21 (DE3) pLysS. Cultures were supplemented during expression with 250 μM ZnCl_2_. Cells were lysed by sonication in lysis buffer containing 20 mM Tris (pH 8.0), 500 mM NaCl, 0.1% NP-40 and cOmplete Protease Inhibitor Cocktail (Roche) and trimeric complexes were affinity purified via 6xHis-tagged RING1B on Ni^2+^-charged IMAC Sepharose 6 Fast Flow resin (GE Healthcare). 10 mM imidazole was added to lysates during binding. Wash buffer contained 50 mM NaH_2_PO_4_ (pH 8.0), 300 mM NaCl and 20 mM imidazole and protein was eluted in wash buffer containing increasing imidazole (100-250 mM). Purified PRC1 complexes were dialysed into BC100 (50 mM HEPES (pH 7.9), 100 mM KCl, 10% Glycerol, 1 mM DTT).

#### Reconstitution of Nucleosomes

Nucleosomes were reconstituted as described previously (Dyer et al., 2004). Recombinant *Xenopus* histones were expressed in *E. coli* BL21(DE3) pLysS and purified from inclusion bodies via Sephacryl S-200 gel filtration (GE Healthcare). Stoichiometric amounts of each core histone were incubated together under high salt conditions (2 M NaCl) and the resulting histone octamer purified using a Superdex 200 gel filtration column (GE Healthcare). Purified 147bp DNA carrying the 601 nucleosome-positioning sequence was a kind gift from the Brockdorff lab. Purified DNA, in slight excess to octamers, was mixed together in 2 M NaCl and diluted stepwise with 10 mM Tris (pH 7.5) to reach a final concentration of 100 mM NaCl. The reconstituted nucleosomes were analyzed on a 0.8% Tris-borate agarose gel and concentrated using a 30,000 MWCO spin concentrator (GE Healthcare).

#### E3 ubiquitin ligase assays

H2A ubiquitylation assays were carried out as previously (Rose et al., 2016). Briefly, UBE1 (Boston Biochem), UbcH5c (Enzo), methylated ubiquitin (Boston Biochem) and ATP (Life technologies) were pre-incubated for 20 min at 37°C prior to addition of reconstituted PRC1 and nucleosomes. Reactions were allowed to proceed for 1 h at 37°C then quenched with 30 mM EDTA and subject to SDS-PAGE for western blot analysis. Western blots were probed with antibodies which recognize Histone H2A in both ubiquitylated and unmodified form (Millipore 07-146) and Histone H3 (CST, 96C10), followed by incubation with LiCOR IRDye secondary antibodies (800CW goat anti-rabbit and 680RD goat anti-mouse). Western blots were imaged using the LiCOR Odyssey Fc imaging system and band intensities were quantified using ImageStudio. H2A band intensities were normalized to H3 and the fraction of ubiquitylated H2A relative to total H2A was quantified. Data were visualized and dose-response curves fitted using GraphPad Prism 7.

#### Genome engineering by CRISPR/Homology-Directed Repair (HDR)

The pSptCas9(BB)-2A-Puro(PX459)-V2.0 vector was obtained from Addgene (#62988) and sgRNAs were designed using the CRISPOR online tool (http://crispor.tefor.net/crispor.py). Targeting constructs with appropriate homology arms were generated by Gibson assembly using the Gibson Assembly Master Mix kit (New England Biolabs), or in the case of single LoxP sites with 150 bp homology arms, purchased from GeneArt Gene Synthesis (Invitrogen). In all instances, targeting constructs were designed such that Cas9 recognition sites were disrupted by the presence of the LoxP site. ESCs (one well of a 6-well plate) were transfected with 0.5 μg of each Cas9 guide, and 2 μg of targeting construct (where appropriate) using Lipofectamine 3000 (ThermoFisher) according to manufacturer’s guidelines. The day after transfection, cells were passaged at a range of densities and subjected to puromycin selection (1 μg/mL) for 48 h to eliminate any non-transfected cells. Approximately one week later, individual clones were isolated, expanded, and PCR-screened for the desired genomic modification.

#### Cell line generation

Constitutive *Ring1b*^*I53A*^ and *Ring1b*^*I53A/D56K*^ ESCs were generated from E14 ESCs using a Cas9 guide specific for the mutation site in the endogenous *Ring1b* gene, and targeting constructs with approximately 1 kb homology arms. The targeting constructs were designed so that as well as introducing the desired mutation into *Ring1b*, an *Msp*I restriction site was also created to enable screening of clones via a PCR and digest approach. Putative homozygote clones were carried forward for RT-PCR and sequencing to verify that the *Ring1b* transcript carried the desired mutation, as well as western blot analysis. An analogous strategy was used to insert the I50A/D53K mutation into *Ring1a* (see below).

To generate the PRC1^CPM^ line, a targeting construct was generated comprising exon 3 of *Ring1b* in forward orientation (flanked by 100 bp of *Ring1b* intron 2/intron 3) followed by a mutant copy of exon 3 (encoding I53A and D56K mutations) in reverse orientation (flanked by splice donor and acceptor sites from mouse *IgE* gene). Both the wild-type and mutant versions of exon 3 were codon optimized at wobble positions to minimize sequence similarity, thereby avoiding hairpin formation and allowing the two to be easily distinguished. This exon 3 pair was flanked by doubly inverted LoxP/Lox2272 sites and approximately 1 kb homology arms (see Table S1 for sequence). To help ensure that the RING1B^CPM^ cassette was inserted correctly, the RING1B^CPM^ targeting construct was transfected into E14 ESCs in combination with three different Cas9 guides specific for the *Ring1b* locus (see Table S1). Correctly targeted homozygous clones were identified by PCR screening, followed by RT-PCR and sequencing to check for splicing defects. CreERT2 was then inserted into the *Rosa26* locus using a *Rosa26*-specific Cas9 guide, and using a similar approach, the I50A/D53K mutation was constitutively knocked into both copies of endogenous *Ring1a.* The final PRC1^CPM^ cell line was validated by PCR, RT-PCR and western blot, with and without tamoxifen treatment.

The isogenic PRC1^CKO^ line used in this study was described previously (Fursova et al., 2019). Briefly, exons 1-3 of *Ring1a* were first deleted using Cas9 guides flanking the 1.5 kb deletion region, and Cre-ERT2 was inserted into the *Rosa26* locus using a *Rosa26*-specific guide (see Table S1). *Ring1a*^*−/−*^*;CreERT2* ESCs were then subjected to two sequential rounds of genome editing to insert parallel LoxP sites flanking exon 2 (the first coding exon) of *Ring1b*.

The control TIR1-only and AID-RING1B lines were generated from E14 ESCs as described previously (Rhodes et al., 2019). Briefly, Cas9 engineering was used to insert the coding sequence for *Oryza sativa* TIR1 into the *Rosa26* locus, thereby generating the TIR1-only control line. To generate the AID-RING1B line, the TIR1-only line was subjected to further rounds of Cas9-mediated engineering to introduce the auxin inducible degron (AID) tag at the N terminus of both copies of *Ring1b,* and constitutively delete *Ring1a* using Cas9 guides flanking exons 1-3. Western blot analysis was used to confirm loss of RING1B protein in AID-RING1B line in response to auxin treatment, and that RING1B levels remained unchanged in auxin-treated TIR1-only control cells.

HaloTag-SUZ12 PRC1^CPM^ and RING1B-HaloTag PRC1^CPM^ lines were generated from PRC1^CPM^ ESCs using CRISPR-Cas9 guides specific for the N terminus of *Suz12* and C- terminus of *Ring1b*, respectively, along with targeting constructs containing the HaloTag flanked by homology arms of at least 750 bp to introduce the HaloTag at both copies of each gene. After selection with puromycin, cells were labeled with 500 nM Halo-TMR, and cells with significantly higher fluorescence than the similarly labeled parental cell line were FACS-selected and plated at low density to allow picking of individual clones. Homozygous knock-in clones were identified by PCR screening, followed by western blot analysis to confirm homozygous tagging and that levels of protein expression were unchanged compared to the parental cell line.

#### Phenotypic characterization of cell lines

For PRC1^CPM^ and PRC1^CKO^ cells, growth curves were performed by plating out 10,000 cells on each well of a 6 well plate. Cells were harvested at 24 h intervals for a period of 6 days, and live cells (not stained by trypan blue) were counted using a Countess II cell counter (Invitrogen). Counts were performed for 6 independent experiments.

For alkaline phosphatase staining, cells were first fixed in 3.7% formaldehyde for 20 min at 4°C. Cells were then stained in AP staining solution (100 mM Tris-Hcl pH 9, 100 mM NaCl, 5 mM MgCl_2_, 0.4 mg/mL Napthol phosphate N-5000, 1 mg/mL Fast Violet B Salt) for 10 min, rinsed with PBS and then distilled water, and air-dried. For each cell line (UNT and OHT-treated), 3 independent experiments were performed and at least 100 colonies were counted on each occasion.

#### Calibrated ChIP-sequencing (cChIP-seq)

For RING1B, SUZ12, JARID2, PCL2, AEBP2, EPOP, PCGF2, PCGF1 and PCGF6, cChIP-seq (Bonhoure et al., 2014, Hu et al., 2015, Orlando et al., 2014) was performed as described previously (Fursova et al., 2019). Briefly, 5 × 10ˆ7 mouse ESCs (untreated or following 72 h OHT treatment) were mixed with 2 × 10ˆ6 human HEK293T cells. Cells were resuspended in 10 mL phosphate buffered saline (PBS) and crosslinked at 25°C with 2 mM DSG (Thermo Scientific) for 45 min, and then with 1% formaldehyde (methanol-free, Thermo Scientific) for a further 15 min. Reactions were quenched with 125 mM glycine. Crosslinked cells were incubated in lysis buffer (50 mM HEPES pH 7.9, 140 mM NaCl, 1 mM EDTA, 10% glycerol, 0.5% NP40, 0.25% Triton X-100) for 10 min at 4°C. Released nuclei were washed (10 mM Tris-HCl pH 8, 200 mM NaCl, 1 mM EDTA, 0.5 mM EGTA) for 5 min at 4°C. Chromatin was then resuspended in 1 mL of sonication buffer (10 mM Tris-HCl pH 8, 100 mM NaCl, 1 mM EDTA, 0.5 mM EGTA, 0.1% Na deoxycholate, 0.5% N-lauroylsarcosine) and sonicated for 30 min using a BioRuptor Pico (Diagenode), shearing genomic DNA to an average size of 0.5 kb. Following sonication, Triton X-100 was added to a final concentration of 1%.

For ChIP, sonicated chromatin was diluted 10-fold in ChIP dilution buffer (1% Triton X-100, 1 mM EDTA, 20 mM Tris-HCl pH 8, 150 mM NaCl) and pre-cleared for 1 h using Protein A agarose beads (Repligen) blocked with 1 mg/mL BSA and 1 mg/mL yeast tRNA. For each ChIP reaction, 1 mL of diluted and pre-cleared chromatin was incubated overnight with the appropriate antibody, anti-RING1B (CST, D22F2, 3 μl), anti-PCGF1 (in-house, 5 μl), anti-PCGF2 (Santa Cruz, sc-10744, 3 μl), anti-PCGF6 (LifeSpan BioSciences LS-C482495, 3 μl), anti-SUZ12 (CST, D39F6, 3 μl), anti-JARID2 (CST D6M9X, 3 μl), anti-PCL2 (GenWay GWB-FA7207, 2 μl), anti-AEBP2 (CST, D7C6X, 10 μl) or anti-EPOP (Active motif, 61753, 5 μl). Antibody-bound chromatin was captured using blocked protein A agarose for 1 h at 4°C and collected by centrifugation. ChIP washes were performed as described previously (Farcas et al., 2012). ChIP DNA was eluted in elution buffer (1% SDS, 0.1 M NaHCO3) and cross-links were reversed overnight at 65°C with 200 mM NaCl and 2 μL RNase A (Sigma). A matched input sample (10% of original ChIP reaction) was identically treated. The following day, ChIP samples and Inputs were incubated with Proteinase K (Sigma) for 1.5 h at 56°C and purified using ChIP DNA Clean and Concentrator Kit (Zymo Research).

cChIP-seq libraries for both ChIP and Input samples were prepared using NEBNext Ultra DNA Library Prep Kit for Illumina, following manufacturer’s guidelines. Samples were indexed using NEBNext Multiplex Oligos. The average size and concentration of all libraries was analyzed using the 2100 Bioanalyzer High Sensitivity DNA Kit (Agilent) followed by qPCR using SensiMix SYBR (Bioline, UK) and KAPA Illumina DNA standards (Roche). Libraries were sequenced as 40 bp paired-end reads on Illumina NextSeq 500 platform.

#### Native cChIP-sequencing

Native cChIP-seq for H2AK119ub1, H3K27me3 and H3K4me3 was performed as described previously (Fursova et al., 2019). Briefly, 5 × 10ˆ7 mouse ESCs (both untreated and following 72 h OHT treatment) were mixed with 2 × 10ˆ7 *Drosophila* SG4 cells in PBS. Mixed cells were pelleted and nuclei were released by resuspending in ice cold lysis buffer (10 mM Tris-HCl pH 8.0, 10 mM NaCl, 3 mM MgCl_2_, 0.1% NP40, 5 mM N-ethylmaleimide). Nuclei were then washed, and resuspended in 1 mL of MNase digestion buffer (10 mM Tris-HCl pH 8.0, 10 mM NaCl, 3 mM MgCl_2_, 0.1% NP40, 0.25 M sucrose, 3 mM CaCl_2_, 10 mM N-ethylmaleimide, 1x protease inhibitor cocktail (Roche)). Each sample was incubated with 200 units of MNase (Fermentas) at 37°C for 5 min, followed by the addition of 4 mM EDTA to halt MNase digestion. Following centrifugation at 1500 g for 5 min at 4°C, the supernatant (S1) was retained. The remaining pellet was incubated with 300 μl of nucleosome release buffer (10 mM Tris-HCl pH 7.5, 10 mM NaCl, 0.2 mM EDTA, 1x protease inhibitor cocktail (Roche), 10 mM N-ethylmaleimide) at 4°C for 1 h, passed five times through a 27G needle using a 1 mL syringe, and spun at 1500 g for 5 min at 4°C. The second supernatant (S2) was collected and combined with corresponding S1 sample from above. A small amount of S1/S2 DNA was purified and visualized on a 1.5% agarose gel to confirm digestion to mostly mono-nucleosomes.

For ChIP experiments, S1/S2 nucleosomes were diluted 10-fold in native ChIP incubation buffer (70 mM NaCl, 10 mM Tris pH 7.5, 2 mM MgCl2, 2 mM EDTA, 0.1% Triton, 1x protease inhibitor cocktail (Roche), 10 mM N-ethylmaleimide (NEM)), and 1 mL aliquots were made. Each ChIP reaction was then incubated overnight at 4°C with the appropriate antibody, 5 μL of anti-H2AK119ub1 (Cell Signaling Technology, D27C4), 5 μL of anti-H3K27me3 (in-house) or 3 μL anti-H3K4me3 (in-house) antibody. Antibody-bound nucleosomes were captured using protein A agarose (Repligen) beads, pre-blocked in native ChIP incubation buffer supplemented with 1 mg/mL BSA and 1 mg/mL yeast tRNA, for 1 h at 4°C and collected by centrifugation. Immunoprecipitated material was washed four times with Native ChIP wash buffer (20 mM Tris pH 7.5, 2 mM EDTA, 125 mM NaCl, 0.1% Triton X-100) and once with Tris-EDTA buffer (10 mM Tris pH 8, 1 mM EDTA). ChIP DNA was eluted using 100 μL of elution buffer (1% SDS, 0.1 M NaHCO3), and then purified using ChIP DNA Clean and Concentrator Kit (Zymo Research). For each individual ChIP sample, DNA from a matched Input control (corresponding to 10% of original ChIP reaction) was also purified. Native cChIP-seq library preparation and sequencing was performed as described above for cChIP-seq.

#### Calibrated RNA-sequencing (cRNA-seq)

For cRNA-seq, 1 × 10ˆ7 mouse ESCs (both untreated and following 72 h OHT treatment) were mixed with 4 × 10ˆ6 *Drosophila* SG4 cells in 600 μL PBS. For RNA extraction, 400 μL of cells was used (corresponding to 6.7 × 10ˆ6 mouse ESCs), and for DNA extraction the remaining 200 μL of cells was used (corresponding to 3.3 × 10ˆ6 mouse ESCs). RNA extraction was performed using RNeasy mini kit columns (QIAGEN) following manufacturer’s guidelines, and 10 μg was subjected to Turbo DNase (ThermoFisher) treatment to remove any contaminating DNA. Quality of RNA was assessed using 2100 Bioanalyzer RNA 6000 Pico kit (Agilent). Next, RNA samples were depleted of rRNA using the NEBNext rRNA Depletion kit (NEB). RNA-seq libraries were prepared using the NEBNext Ultra II Directional RNA Library Prep kit (NEB). To quantitate the consistency of spike-in cell mixing for each individual sample, a matched sample of cells was used to isolate genomic DNA using Quick-DNA miniprep kit (Zymo). Libraries from gDNA were prepared using NEBNext Ultra II FS kit (NEB) following manufacturer’s guidelines. RNA and DNA libraries were sequenced as 80 bp paired-end reads on the Illumina NextSeq 500 platform.

#### Preparation of nuclear and histone extracts and immunoblotting

For nuclear extraction, ESCs were washed with PBS and then resuspended in 10 volumes of Buffer A (10 mM HEPES pH 7.9, 1.5 mM MgCl2, 10 mM KCl, 0.5 mM DTT, 0.5 mM PMSF and protease inhibitor cocktail (Roche)). After 10 min incubation on ice, cells were recovered by centrifugation at 1500 g for 5 min and resuspended in 3 volumes of Buffer A supplemented with 0.1% NP-40. The released nuclei were pelleted by centrifugation at 1500 g for 5 min, followed by resuspension in 1 volume of Buffer C (5 mM HEPES (pH 7.9), 26% glycerol, 1.5 mM MgCl 2, 0.2 mM EDTA, protease inhibitor cocktail (Roche) and 0.5 mM DTT) supplemented with 400 mM NaCl. The extraction was allowed to proceed on ice for 1 h with occasional agitation, then the nuclei were pelleted by centrifugation at 16,000 g for 20 min at 4°C. The supernatant was taken as the nuclear extract.

For histone extraction, ESCs were washed with RSB supplemented with 20 mM NEM, incubated on ice for 10 min in RSB with 0.5% NP-40 and 20 mM NEM, pelleted by centrifugation at 800 g for 5 min and incubated in 2.5 mM MgCl2, 0.4 M HCl and 20 mM NEM on ice for 30 min. After that, cells were pelleted by centrifugation at 16,000 g at 4°C for 20 min, the supernatant recovered and precipitated on ice with 25% TCA for 30 min, followed by centrifugation at 16,000 g for 15 min at 4°C to recover histones. Following two acetone washes, the histones were resuspended in 150 μL 1xSDS loading buffer and boiled at 95°C for 5 min. Finally, any insoluble precipitate was pelleted by centrifugation at 16,000 g for 15 min at 4°C and the soluble fraction retained as the histone extract. Histone concentrations across samples were compared by Coomassie Blue staining following SDS-PAGE. Semiquantitative western blot analysis of histone extracts was performed using LI-COR IRDye® secondary antibodies and imaging was done using the LI-COR Odyssey Fc system. To measure the changes in bulk H2AK119ub1 levels, the relative signal of H2AK119ub1 to H3 or H4 histones was quantified.

#### Co-immunoprecipitation

For co-immunoprecipitation reactions, 400 μg of nuclear extract from wild-type or RING1B^I53A/D56K^ ESCs was added to BC150 buffer (150 mM KCl, 10% glycerol, 50 mM HEPES (pH 7.9), 0.5 mM EDTA, 0.5 mM DTT) with 1x protease inhibitor cocktail (Roche) to a total volume of 550 μl. A 50 μL Input sample was retained, and 5 μg of mouse monoclonal anti-RING1B antibody (Atsuta et al., 2001) was added to the remaining 500 μL of sample. Immunoprecipitation reactions were then incubated overnight at 4°C. Immunoprecipitated material was collected with Protein A agarose beads and washed four times in 1 mL of BC150 buffer. Following the final wash step, beads were directly resuspended in 100 μL of 1x SDS loading buffer (2% SDS, 0.1 M Tris pH 6.8, 0.1 M DTT, 10% glycerol, 0.1% bromophenol blue) and placed at 95°C for 5 min. 1x SDS loading buffer was similarly added to Input samples which were also incubated at 95°C for 5 min, prior to SDS-PAGE and western blot analysis.

#### Polycomb body imaging

To image Polycomb bodies in live cells, HaloTag-SUZ12;PRC1^CPM^ or RING1B-HaloTag;PRC1^CPM^ cells were plated on gelatinised 35 mm Petri dish, 14 mm Microwell 1.5 coverglass dishes (MatTek, #P35G-1.5-14-C) at least 5 h before imaging. Prior to imaging, cells were labeled with 500 nm JF_549_ (Grimm et al., 2017) for 15 min at 37°C, followed by 3 washes, changing medium to Fluorobrite DMEM (Thermo Fisher Scientific) supplemented as described for general ESC culture above. Cells were incubated for a further 30 min in supplemented Fluorobrite DMEM with 10 μg/mL Hoechst 33258 (Thermo Fisher Scientific) at 37°C and washed once more before imaging. Cells were imaged on an IX81 Olympus microscope connected to a Spinning Disk Confocal system (UltraView VoX PerkinElmer) using an EMCCD camera (ImagEM, Hamamatsu Photonics) in a 37°C heated, humidified, CO_2_-controlled chamber. Z stacks were acquired using a 100x PlanApo NA 1.40 oil-immersion objective heated to 37°C, using Volocity software (PerkinElmer). HaloTag-JF_549_ was imaged with a 561 nm laser at 1.25 s exposure at 15% laser power, while Hoechst was imaged with a 405 nm laser at 250 ms exposure at 20% laser power. Z stacks were acquired at 150 nm intervals.

#### Capture-C library preparation

CaptureC libraries were prepared as described previously (Hughes et al., 2014). Briefly, 10^6^ mouse ESCs were trypsinized, collected in 50 mL falcon tubes in 9.3 mL media and crosslinked with 1.25 mL 16% formaldehyde for 10 min at room temperature. Cells were quenched with 1.5 M glycine, washed with PBS and lysed for 20 min at 4°C lysis buffer (10mM Tris pH 8, 10 mM NaCl, 0.2% NP-40, supplemented with complete proteinase inhibitors) prior to snap freezing in 1 mL lysis buffer at −80°C. Lysates were then thawed on ice, pelleted and resuspended in 650 μl 1x *Dpn*II buffer (NEB). Three 1.5ml tubes with 200μl lysate each were treated in parallel with SDS (0.28% final concentration, 1 h, 37°C, interval shaking 500 rpm, 30 s on/30 s off), quenched with trypsin (1.67% final concentration, 1 h, 37°C, interval shaking 500rpm, 30 s on/30 s off) and subjected to a 24 h digestion with 3x10μl DpnII (homemade, 37°C, interval shaking 500rpm, 30 s on/30 s off). Each chromatin aliquot was independently ligated with 8 μl T4 Ligase (240 U) in a volume of 1440 μl (20 h, 16°C). Following this, the nuclei containing ligated chromatin were pelleted to remove any non-nuclear chromatin, reverse-crosslinked and the ligated DNA was phenol-chloroform purified. The sample was resuspended in 300 μl water and sonicated 13x (Pico Bioruptor, 30 s on, 30 s off) or until a fragment size of approximately 200 bp was reached. Fragments were size-selected using AmpureX beads (Beckman Coulter: A63881, ratios: 0.85x / 0.4x). 2x 1-5 μg of DNA were adaptor ligated and indexed using the NEBNext DNA library Prep Reagent Set (New England Biolabs: E6040S/L) and NEBNext Multiplex Oligos for Illumina Primer sets 1 (New England: E7335S/L) and 2 (New England: E7500S/L). The libraries were amplified 7x using Herculase II Fusion Polymerase kit (Agilent: 600677).

#### Capture-C hybridization and sequencing

5′ biotinylated probes were designed using the online tool by the Hughes lab (CapSequm) to be 70-120bp long and two probes for each promoter of interest. The probes were pooled at 2.9 nM each. Samples were captured twice and hybridizations were carried out for 72 h and for 24 h for the first and the second captures, respectively. To even out capture differences between tubes, libraries were pooled prior to hybridization at 1.5 μg each. Hybridization was carried out using Nimblegen SeqCap (Roche, Nimblegen SeqCap EZ HE-oligo kit A, Nimblegen SeqCap EZ HE-oligo kit B, Nimblegen SeqCap EZ Accessory kit v2, Nimblegen SeqCap EZ Hybridization and wash kit) following manufacturer’s instructions for 72 h followed by a 24 h hybridization (double Capture). The captured library molarity was quantified by qPCR using SensiMix SYBR (Bioline, UK) and KAPA Illumina DNA standards (Roche) and sequenced on Illumina NextSeq 500 platform as 80 bp paired-end reads for three biological replicates.

### Quantification and Statistical Analysis

#### Massive parallel sequencing, data processing and normalization

For calibrated ChIP-seq, paired-end reads were aligned to the concatenated mouse and spike-in genome sequences (mm10+dm6 for native cChIP-seq, and mm10+hg19 for cross-linked cChIP-seq) using Bowtie 2 (Langmead and Salzberg, 2012) with the “–no-mixed” and “–no-discordant” options. Only uniquely mapped reads after removal of PCR duplicates with Sambamba (Tarasov et al., 2015) were used for downstream analysis.

For cRNA-seq, first, paired-end reads were aligned using Bowtie 2 (with “–very-fast,” “–no-mixed” and “–no-discordant” options) against the concatenated mm10 and dm6 rDNA genomic sequence (GenBank: BK000964.3 and M21017.1) and reads mapping to rDNA were discarded. All unmapped reads were then aligned against the concatenated mm10 and dm6 genome sequences using STAR (Dobin et al., 2013). Finally, reads that failed to map using STAR were aligned against the mm10+dm6 concatenated genome using Bowtie 2 (with “–sensitive-local,” “–no-mixed” and “–no-discordant” options) to improve mapping of introns. Uniquely aligned reads from the last two steps were combined for further analysis. PCR duplicates were removed using Sambamba (Tarasov et al., 2015). For the corresponding gDNA-seq experiments, paired-end read alignment and processing was carried out as described above for cChIP-seq.

For Capture-C, paired-end reads were aligned and filtered for HiC artifacts using HiCUP (Wingett et al., 2015) and Bowtie2 (Langmead and Salzberg, 2012) with fragment filter set to 100-800bp. A list of all Next-Generation sequencing experiments carried out in this study and the number of uniquely aligned reads in each experiment can be found in Table S2.

For visualization of cChIP-seq and cRNA-seq data and annotation of genomic regions with read counts, uniquely aligned mouse reads were normalized using dm6 or hg19 spike-in as described previously (Fursova et al., 2019). Briefly, mm10 reads were randomly subsampled based on the total number of spike-in (dm6 or hg19) reads in each sample. To account for any minor variations in spike-in cell mixing between replicates, we used the ratio of spike-in/mouse total read counts in the corresponding Input/gDNA-seq samples to correct the subsampling factors. After normalization, read coverages for individual biological replicates were compared across RING1B peaks for cChIP-seq or gene bodies for cRNA-seq using multiBamSummary and plotCorrelation from deepTools (Ramírez et al., 2014). For each experimental condition, biological replicates correlated well (Pearson correlation coefficient > 0.9, see Table S3) and were merged for downstream analysis. Genome coverage tracks were generated using the pileup function from MACS2 (Zhang et al., 2008) for cChIP-seq and genomeCoverageBed from BEDTools (Quinlan, 2014) for cRNA-seq and visualized using the UCSC genome browser (Kent et al., 2002). BigwigCompare from deeptools (v3.1.1) (Ramírez et al., 2014) was used to make differential genome coverage tracks.

#### Peak calling

To identify genomic regions bound by RING1B, SUZ12 and PCGF2, we carried out peak calling using MACS2 (“BAMPE” and “–broad” options specified), with corresponding Input samples used as a control. For RING1B and SUZ12, peaks were called using merged biological replicates from untreated PRC1^CPM^ and PRC1^CKO^ cells for RING1B and SUZ12 cChIP-seq respectively, and only peaks identified in both cell lines were selected. For PCGF2, individual replicates from untreated PRC1^CPM^ ESC were used, and only peaks identified in all biological replicates were taken forward. For all peak sets, peaks overlapping with a custom-build set of blacklisted genomic regions were discarded to remove sequencing artifacts. For RING1B-bound regions, RING1B cChIP-seq in PRC1^CKO^ ESCs was used to filter out peaks which showed no significant loss of RING1B signal following tamoxifen treatment (p-adj < 0.05 and > 2-fold). In total, we were able to identify 18643 RING1B peaks, 7438 SUZ12 peaks and 3680 PCGF2 peaks. Using the overlap between these peak sets, we defined Polycomb (PcG)-occupied regions as RING1B peaks that overlap SUZ12 peaks (n = 7074), and PCGF2 target sites as RING1B peaks overlapping PCGF2 peaks (n = 3568). To characterize low-level genomic blanket of H2AK119ub1, we have generated a set of 100 kb windows spanning the genome (n = 27,348) using makewindows function from BEDtools (v2.17.0).

#### Read count quantitation and analysis

For cChIP-seq, computeMatrix and plotProfile/plotHeatmap from deeptools were used to perform metaplot and heatmap analysis of read density at regions of interest. For each cChIP-seq dataset and each cell line, read density at the peak center in untreated cells was set to 1. For chromosome-wide density plots, read coverage in 250 kb bins was calculated using a custom R script utilizing GenomicRanges, GenomicAlignments and Rsamtools Bioconductor packages (Huber et al., 2015) and visualized using ggplot2. For cChIP-seq, target regions of interest were annotated with read counts using multiBamSummary from deeptools (“–outRawCounts”). For comparative boxplot analysis, read counts from merged spike-in normalized replicates were used, while for differential enrichment analysis, read counts from individual biological replicates prior to spike-in normalization were obtained. For differential gene expression analysis, we used a custom Perl script utilizing SAMtools to obtain read counts for a custom-built non-redundant mm10 gene set (n = 20633), derived from mm10 refGene genes by removing very short genes with poor sequence mappability and highly similar transcripts.

Normalized read counts and log2 fold changes for different genomic intervals were visualized using custom R scripts and ggplot2. For boxplot analysis of cChIP-seq signal for different factors before and after treatment, read counts were normalized to the genomic region size (in kb) and median value of cChIP-seq signal in untreated cells, log2 of which was set to 1. For boxplots comparing H2AK119ub1 enrichment at RING1B-bound sites and 100 kb genomic windows, read density is shown relative to the signal at RING1B-bound sites in untreated cells. For boxplots, boxes show interquartile range (IQR) and whiskers extend by 1.5xIQR. To study the relationship between the levels of different factors/histone modifications and gene expression at RING1B-bound genomic regions, the RING1B target sites that overlapped with gene promoters were divided into percentiles based on the expression level of the associated gene in untreated PRC1^CPM^ cells. For each percentile, mean read density normalized to the genomic region size (in kb) was plotted relative to the mean read density in the fiftieth percentile, together with loess regression trendlines. ggcor function from the GGally (v1.4.0) R package was used to generate a correlation matrix for the association of RING1B occupancy before and after tamoxifen treatment in PRC1^CPM^ cells with a set of chromatin features and occupancy of other Polycomb factors. All correlation analyses in the paper used Pearson correlation coefficient to measure the strength of the association between the variables and were visualized using scatterplots colored by density with stat_density2d. Linear regression lines were plotted using stat_poly_eq function from the ggpmisc (v0.3.1) R package, together with the model’s R^2^ coefficient of determination. Student’s t test and Wilcoxon rank sum statistical tests were also performed in R with samples considered to be independent and two-sided alternative hypothesis, unless otherwise specified.

For CaptureC, read counts and interaction scores (significance of interactions) for the captured gene promoters were obtained using the Bioconductor package CHiCAGO (Cairns et al., 2016). For visualization of CaptureC data, weighted read counts from CHiCAGO data files for merged biological replicates were normalized to the total number of reads aligning to the captured gene promoters and further to the number of promoters in the respective CaptureC experiment. Bigwig files were generated from these normalized read counts. For comparative boxplot analysis, interactions called by CHiCAGO (score > = 5) across all samples were aggregated and interactions with a distance of less than 10 DpnII fragments were merged to a single interaction peak. For each interaction peak, we then quantified mean normalized read count and CHiCAGO scores of all overlapping *Dpn*II fragments.

#### Differential cChIP-seq enrichment and gene expression analysis

To identify significant changes in cChIP-seq and cRNA-seq, we used a custom R script that incorporates spike-in calibration into DESeq2 analysis (Love et al., 2014). In order to do this, we used read counts from the spike-in genome at a control set of intervals to calculate DESeq2 size factors for normalization of raw mm10 read counts (as has been previously described in (Fursova et al., 2019, Taruttis et al., 2017). Unique dm6 genes from refGene were used for spike-in normalization of cRNA-seq, while 10 kb (for hg19 spike-in) or 1 kb (for dm6 spike-in) windows spanning the genome were used for differential enrichment analysis of cChIP-seq. Prior to quantification, spike-in reads were pre-normalized using the spike-in/mouse read ratio derived from the corresponding Input or genomic DNA-seq sample in order to account for minor variations in mixing of mouse and spike-in cells. For a change to be called significant, we applied a threshold of p-adj < 0.05 and fold change > 1.5, unless otherwise specified. Log2-fold change values were visualized using R and ggplot2 with MA plots and violin plots. For MA plots, density of the data points across y axis is shown to reflect the general direction of gene expression changes.

#### Annotation of Polycomb target genes

Mouse genes in a custom non-redundant set (n = 20633) that was used for differential gene expression analysis were classified into three groups based on the overlap of their gene promoters with non-methylated CpG islands (NMI), as well as RING1B- and SUZ12- bound sites. NMIs (n = 27047) were identified using MACS2 peak calling from BioCAP-seq data (Long et al., 2013) as regions which are enriched with non-methylated CpG-rich DNA. All genes with promoters (TSS ± 2500 bp) not overlapping with NMIs were referred to as non-NMI genes (n = 5326). Genes that contained NMIs at their promoters were further sub-divided into PcG-occupied genes (n = 6283), if their promoters also overlapped with both RING1B and SUZ12-bound sites defined in this study, and non-PcG-occupied genes (n = 9024), if they did not. For genes with several described transcripts and promoters, a complete mm10 refGene gene set was used for classification, with the overlap of at least one promoter with the feature of interest being required to assign a gene into PcG/non-PcG occupied categories. Finally, we refer to a subset of PcG-occupied genes which showed a statistically significant increase in gene expression following removal of PRC1 in PRC1^CKO^ cells, as PRC1-repressed genes (n = 2482).

In order to identify genes that were differentially affected by the removal of PRC1 or specifically PRC1 catalytic activity, we used two complementary approaches. First, we isolated genes that were expressed at significantly lower levels (p-adj < 0.05 and fold change > 1.5) in PRC1^CPM^ as compared to PRC1^CKO^ cells following tamoxifen treatment. In addition, we also identified genes, for which the magnitude of expression changes following tamoxifen treatment was significantly smaller in PRC1^CPM^ cells than in PRC1^CKO^ cells, using the DESeq2 design that included the interaction term between the cell line and treatment factors. Combination of these two approaches has yielded a total of 241 PRC1-repressed genes that were derepressed to a smaller extent in PRC1^CPM^ cells as compared to PRC1^CKO^ cells following tamoxifen treatment.

#### Analysis of live-cell imaging of Polycomb bodies

To segment Polycomb bodies in individual live cells for analysis, nuclei were first manually segmented based on Hoechst fluorescence using TANGO in ImageJ (Ollion et al., 2013). 561 nm channels of z stacks were deconvolved using Olympus cellSens software (constrained iterative deconvolution, 5 cycles). Deconvolved 561 nm z stacks were masked using outputs from TANGO, and individual Polycomb bodies identified using a custom script. Briefly, segmented nuclei were background subtracted using a 4 px rolling ball and a mask of Polycomb bodies generated using Otsu thresholding. 3D Objects Counter in ImageJ was used to quantify the properties of the masked Polycomb bodies, and its outputs were processed and analyzed using R.

### Data and Code Availability

The high-throughput data reported in this study have been deposited in GEO under the accession number GEO: GSE132754. Published data used in this study include BioCAP-seq (GEO: GSE43512 (Long et al., 2013)); MAX ChIP-seq (GEO: GSE48175; (Krepelova et al., 2014)); H2AK119ub1, H3K27me3 and RING1B cChIP-seq together with corresponding Inputs in PRC1^CKO^ ESCs (GEO: GSE119618; (Fursova et al., 2019)); cRNA-seq in *Pcgf4*^*−/−*^*4;Pcgf2*^*fl/fl*^ (GEO: GSE119619; (Fursova et al., 2019)); CaptureC in RING1B^deg^ and control cell lines (Rhodes et al., 2019), and 4sU RNA-seq gene expression data for mESCs following RA-induced differentiation (GEO: GSE98756) (Dimitrova et al., 2018). All R and Perl scripts used for data analysis in this study are available upon request.

## Supplemental Information

Document S1. Figures S1–S7

Table S1. A List of DNA Sequences for Target Constructs and CRISPR-Cas9 Guides Used to Generate Mouse Embryonic Stem Cell Lines Described in the Paper, Related to STAR Methods

Table S2. A List of All Next-Generation Sequencing Experiments Performed in This Study Detailing the Number of Uniquely Aligned Reads for Mouse and Spike-In Genomes, Related to STAR Methods

Table S3. Summary of Pairwise Correlations between Individual Biological Replicates for All Next-Generation Sequencing Experiments Performed in This Study, Related to STAR Methods

Document S2. Article plus Supplemental Information
