# Supplementary material for: PRC1 Catalytic Activity Is Central to Polycomb System Function
Source: Mol Cell. 2020 Feb 20;77(4):857–874.e9. doi: 10.1016/j.molcel.2019.12.001 (PMC7033600; doi:10.1016/j.molcel.2019.12.001)
Supplement: Document S1. Figures S1–S7 [file mmc1.pdf]

Molecular Cell, Volume *77*

Supplemental Information

PRC1 Catalytic Activity Is Central to Polycomb System Function

Neil P. Blackledge, Nadezda A. Fursova, Jessica R. Kelley, Miles K. Huseyin, Angelika Feldmann, and Robert J. Klose

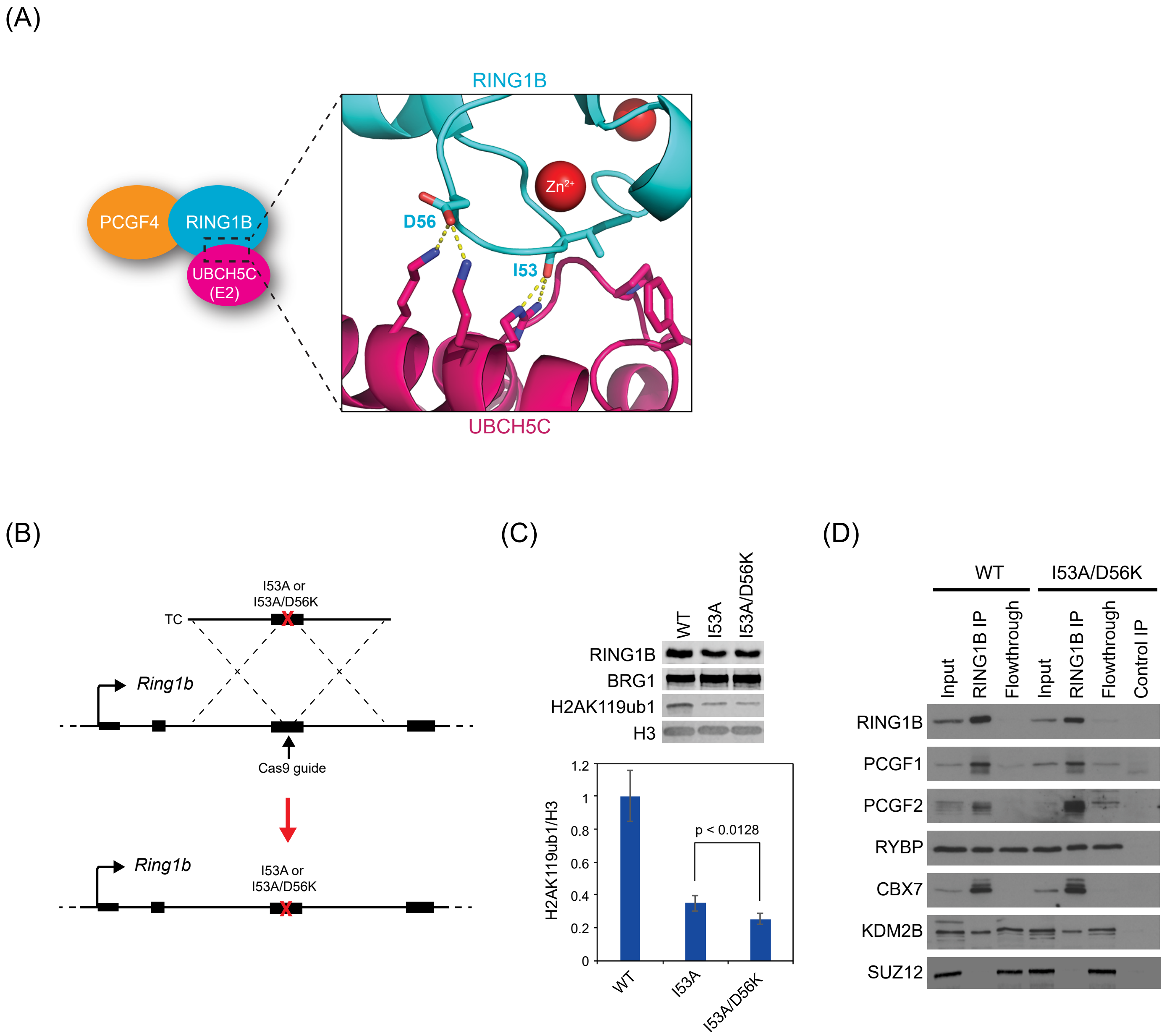
(A)
RING1B
Zn2+
D56
I53
PCGF4
RING1B
UBCH5C
(E2)
UBCH5C
(B)
I53A or
I53A/D56K
TC
Ring1b
Cas9 guide
Ring1b
I53A or
I53A/D56K
(C)
WT
I53A
I53A/D56K
RING1B
BRG1
H2AK119ub1
H3
H2AK119ub1/H3
p < 0.0128
1.2
1
0.8
0.6
0.4
0.2
0
WT
I53A
I53A/D56K
(D)
WT
I53A/D56K
Input
RING1B IP
Flowthrough
Input
RING1B IP
Flowthrough
Control IP
RING1B
PCGF1
PCGF2
RYBP
CBX7
KDM2B
SUZ12

Figure S1

Figure S1. Related to Figure 1.

- **(A)** A schematic of the crystal structure illustrating the RING1B-PCGF4 dimer bound to E2 ubiquitin conjugating enzyme (UBCH5C) (PDB ID: 3RPG) (Bentley et al., 2011). The key amino acids that mediate RING1B-E2 interaction are indicated.
- **(B)** A schematic illustrating how I53A or I53A/D56K mutations were introduced into the endogenous *Ring1b* gene in mouse ESCs (TC = targeting construct).
- **(C)** Western blot analysis of RING1B (with BRG1 as a loading control) and H2AK119ub1 (with H3 as a loading control) in RING1B^{WT}, RING1B^{I53A} and RING1B$^{I53A/D56K}$ ESCs (*top panel*). Quantification of H2AK119ub1 levels from the western blot analysis above (*bottom panel*). Error bars show SEM (n = 6) and the p-value denotes statistical significance calculated by a paired one-tailed Student's t-test.
- **(D)** Immunoprecipitation of RING1B from RING1B^{WT} or RING1B$^{I53A/D56K}$ ESCs followed by western blot for cPRC1 and vPRC1 components as indicated. Western blot for SUZ12 (a PRC2 component) was used as a negative control. For RING1B$^{I53A/D56K}$ ESCs, a control IP was performed with an isotype control antibody.

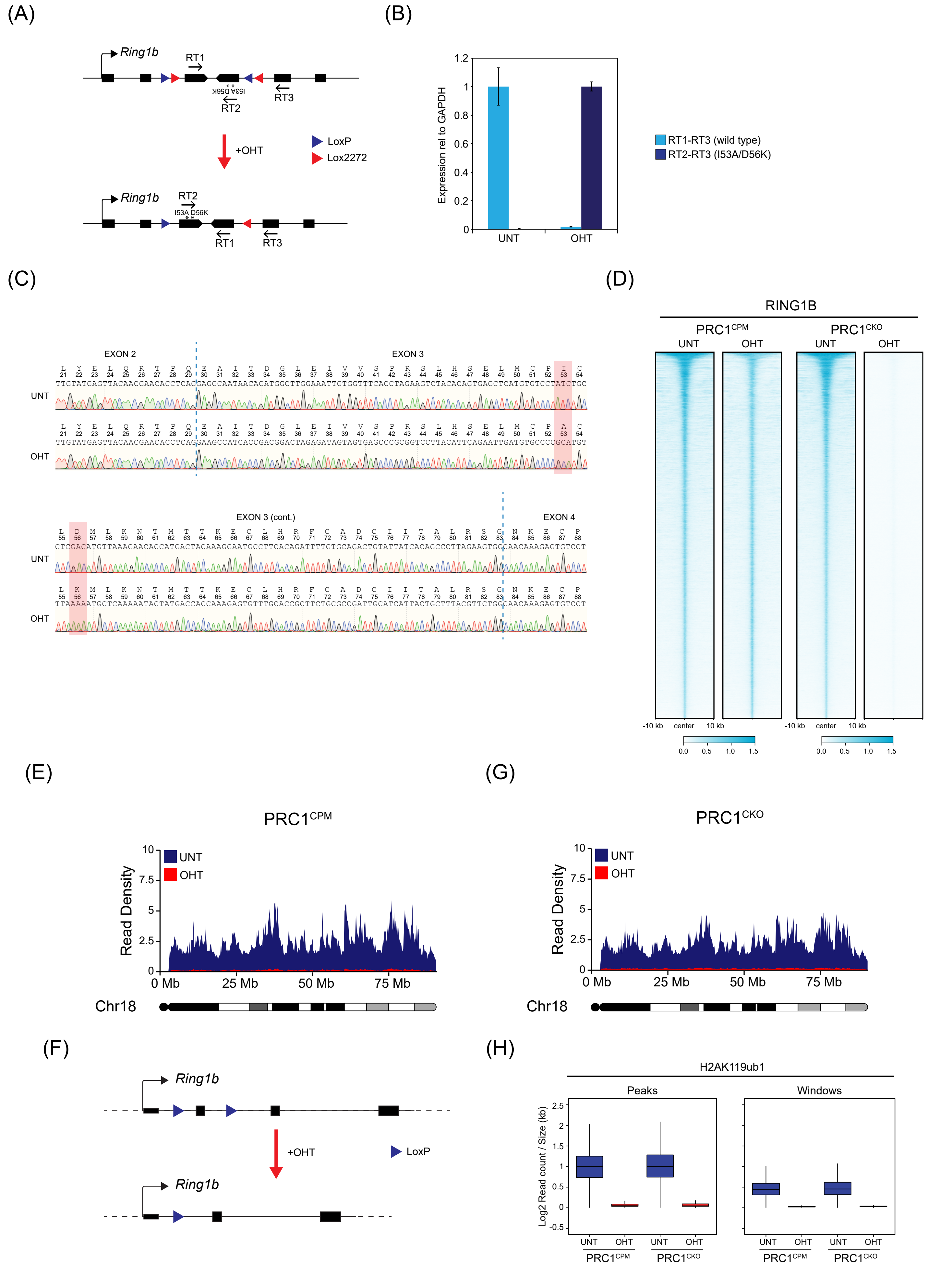

(A)
Ring1b
RT1
I53A D56K
RT2
RT3
+OHT
LoxP
Lox2272
Ring1b
RT2
I53A D56K
RT1
RT3
(B)
Expression rel to GAPDH
UNT
OHT
RT1-RT3 (wild type)
RT2-RT3 (I53A/D56K)
(C)
EXON 2
EXON 3
UNT
OHT
EXON 3 (cont.)
EXON 4
UNT
OHT
(D)
RING1B
PRC1CPM
PRC1CKO
UNT
OHT
UNT
OHT
-10 kb
center
10 kb
(E)
PRC1CPM
Read Density
UNT
OHT
0 Mb
25 Mb
50 Mb
75 Mb
Chr18
(G)
PRC1CKO
Read Density
UNT
OHT
0 Mb
25 Mb
50 Mb
75 Mb
Chr18
(F)
Ring1b
+OHT
LoxP
Ring1b
(H)
H2AK119ub1
Peaks
Windows
Log2 Read count / Size (kb)
UNT
OHT
UNT
OHT
PRC1CPM
PRC1CKO

Figure S2

Figure S2. Related to Figure 2.

- **(A)** A schematic of the endogenous *Ring1b* allele in PRC1^{CPM} cell line before and after addition of OHT, showing positions of the primers used for RT-qPCR quantification of conversion from *Ring1b*^{WT} to *Ring1b*$^{I53A/D56K}$. In untreated cells, incorporation of wild-type exon 3 into *Ring1b* mRNA gives RT-qPCR signal from the primer pair RT1-RT3, but not RT1-RT2. Following OHT treatment and flipping of the exon 3 cassette, incorporation of I53A/D56K version of exon 3 into *Ring1b* mRNA gives RT-qPCR signal from the primer pair RT2-RT3, but not RT1-RT3.
- **(B)** RT-qPCR validation of PRC1^{CPM} line using primers described in (A). Error bars represent SEM (n=4).
- **(C)** DNA sequencing traces of *Ring1b* mRNA in untreated and OHT-treated PRC1^{CPM} cells, showing complete conversion from RING1B^{WT} to RING1B$^{I53A/D56K}$. Vertical dashed lines indicate boundaries between exons. Amino acid sequences are shown above corresponding DNA sequence, with I53A and D56K positions highlighted in red. Wild-type and I53A/D56K mutant versions of exon 3 were engineered to be different at wobble position of each triplet codon, to allow each exon to be easily distinguished and minimize formation of secondary RNA structures that could impact on splicing.
- **(D)** Heatmap analysis of cChIP-seq for RING1B at RING1B-bound sites in PRC1^{CPM} and PRC1^{CKO} cells. The genomic intervals were sorted based on RING1B occupancy in untreated PRC1^{CKO} ESCs.
- **(E)** A chromosome density plot showing H2AK119ub1 cChIP-seq across chromosome 18 in untreated (blue) and OHT-treated (red) PRC1^{CPM} cells.
- **(F)** A schematic of the endogenous *Ring1b* allele in PRC1^{CKO} cell line before and after addition of OHT, showing parallel LoxP sites flanking exon 2 (the first coding exon). OHT treatment causes CRE-mediated deletion of exon 2 which puts the rest of the *Ring1b* coding sequence out of frame, resulting in no functional protein being produced.
- **(G)** A chromosome density plot showing H2AK119ub1 cChIP-seq data across chromosome 18 in untreated (blue) and OHT-treated (red) PRC1^{CKO} cells.
- **(H)** Box plots comparing the normalised H2AK119ub1 cChIP-seq signal at RING1B-bound sites and 100 kb genomic windows in PRC1^{CPM} and PRC1^{CKO} cells.

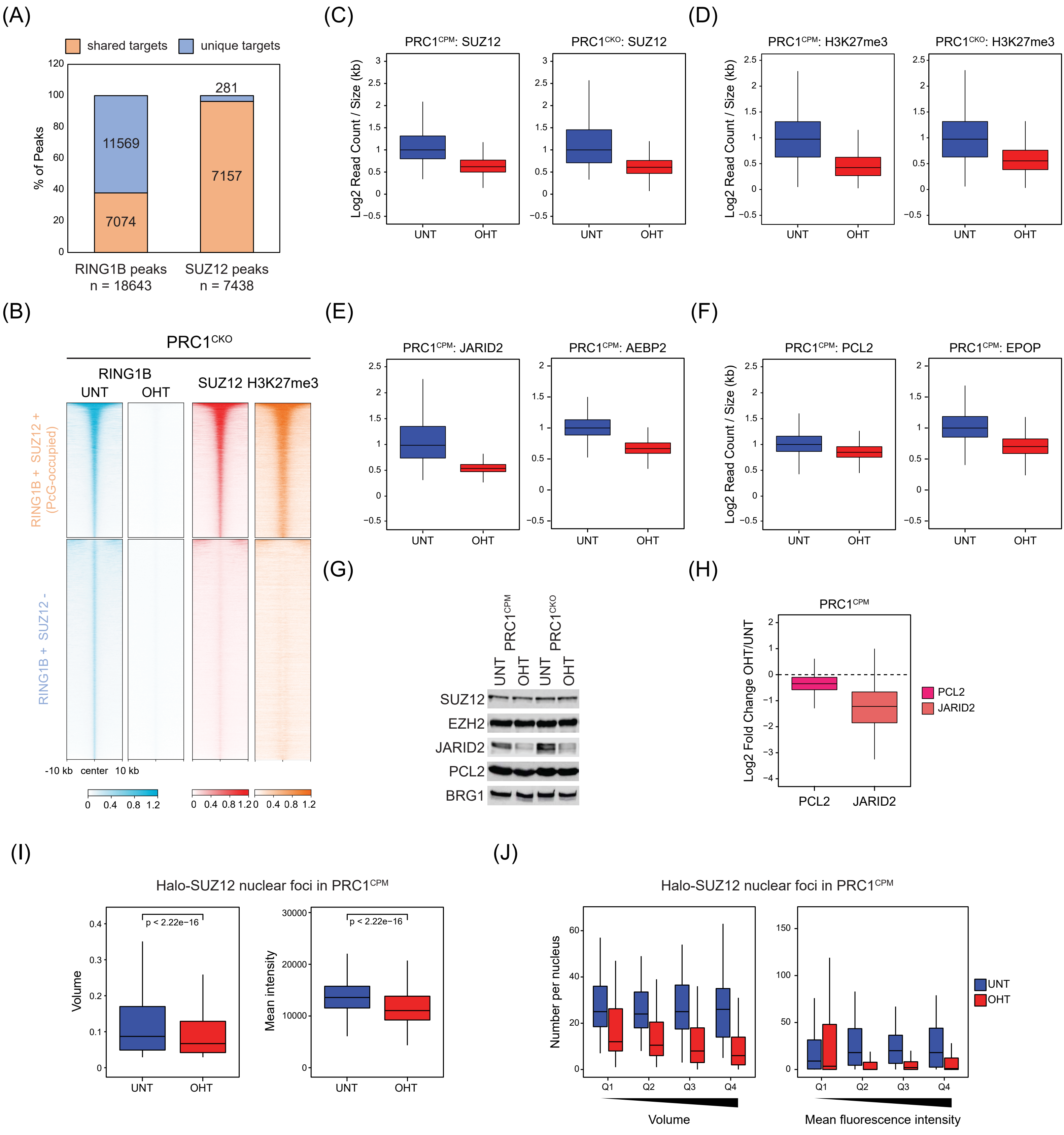
(A)
shared targets
unique targets
% of Peaks
11569
7074
281
7157
RING1B peaks
n = 18643
SUZ12 peaks
n = 7438
(B)
PRC1CKO
RING1B
UNT
OHT
SUZ12 H3K27me3
RING1B + SUZ12 +
(PcG-occupied)
RING1B + SUZ12 -
-10 kb center 10 kb
(C)
PRC1CPM: SUZ12
PRC1CKO: SUZ12
Log2 Read Count / Size (kb)
UNT
OHT
(D)
PRC1CPM: H3K27me3
PRC1CKO: H3K27me3
(E)
PRC1CPM: JARID2
PRC1CPM: AEBP2
(F)
PRC1CPM: PCL2
PRC1CPM: EPOP
(G)
PRC1CPM
PRC1CKO
SUZ12
EZH2
JARID2
PCL2
BRG1
(H)
PRC1CPM
Log2 Fold Change OHT/UNT
PCL2
JARID2
(I)
Halo-SUZ12 nuclear foci in PRC1CPM
p < 2.22e−16
Volume
Mean intensity
(J)
Halo-SUZ12 nuclear foci in PRC1CPM
Number per nucleus
Q1
Q2
Q3
Q4
Volume
Mean fluorescence intensity

Figure S3

Figure S3. Related to Figure 3.

(A) Bar plots showing the overlap between RING1B- and SUZ12-bound genomic regions. This illustrates that PRC1 and PRC2 share a large number of target sites, with > 95% of SUZ12 peaks overlapping with RING1B peaks, while RING1B binding appears to be more broad.

(B) Heatmap analysis of cChIP-seq in $PRC1^{CKO}$ ESCs for RING1B , SUZ12 and H3K27me3 (untreated) at RING1B-bound sites divided into two groups based on the overlap with SUZ12 peaks (RING1B/SUZ12-bound or PcG-occupied (n = 7074) and only RING1B-bound (n = 11,569)). For each group, the genomic regions were sorted based on RING1B occupancy in untreated cells.

(C) Box plots of the normalised cChIP-seq signal for SUZ12 at PcG-occupied sites for untreated and OHT-treated $PRC1^{CPM}$ and $PRC1^{CKO}$ cells.

(D) Box plots of the normalised cChIP-seq signal for H3K27me3 at PcG-occupied sites for untreated and OHT-treated $PRC1^{CPM}$ and $PRC1^{CKO}$ cells.

(E) Box plots of the normalised cChIP-seq signal for PRC2.2-specific subunits (JARID2 and AEBP2) at PcG-occupied sites for untreated and OHT-treated $PRC1^{CPM}$ cells.

(F) Box plots of the normalised cChIP-seq signal for PRC2.1-specific subunits (PCL2 and EPOP) at PcG-occupied sites for untreated and OHT-treated $PRC1^{CPM}$ cells.

(G) Western blot analysis of PRC2 subunits (SUZ12, EZH2, JARID2 and PCL2) in untreated and OHT-treated $PRC1^{CPM}$ ESCs. BRG1 was used as a loading control.

(H) Box plot of log2 fold changes in PCL2 and JARID2 binding at PcG-occupied sites following OHT treatment in $PRC1^{CPM}$ cells.

(I) Box plots comparing volumes (*left*) and mean intensities (*right*) of JF_{549}-Halo-SUZ12 nuclear foci in $PRC1^{CPM}$ ESCs before (n_{cells} = 55) and after OHT treatment (n_{cells} = 52). Cells were counted across two independent experiments. P-values denote statistical significance calculated by a Wilcoxon rank sum test.

(J) Box plots comparing numbers of JF_{549}-Halo-SUZ12 nuclear foci detected in $PRC1^{CPM}$ ESCs before (n_{ells} = 55) and after OHT treatment (n_{cells} = 52), with foci divided into quartiles based on foci volume (*left*) or mean intensities (*right*) in untreated cells (from lowest Q1 to highest Q4). Cells from two independent experiments were analysed. This shows that in the absence of PRC1 catalysis, SUZ12 nuclear foci of different volumes and intensities are lost, with largest and brightest foci being affected the most.

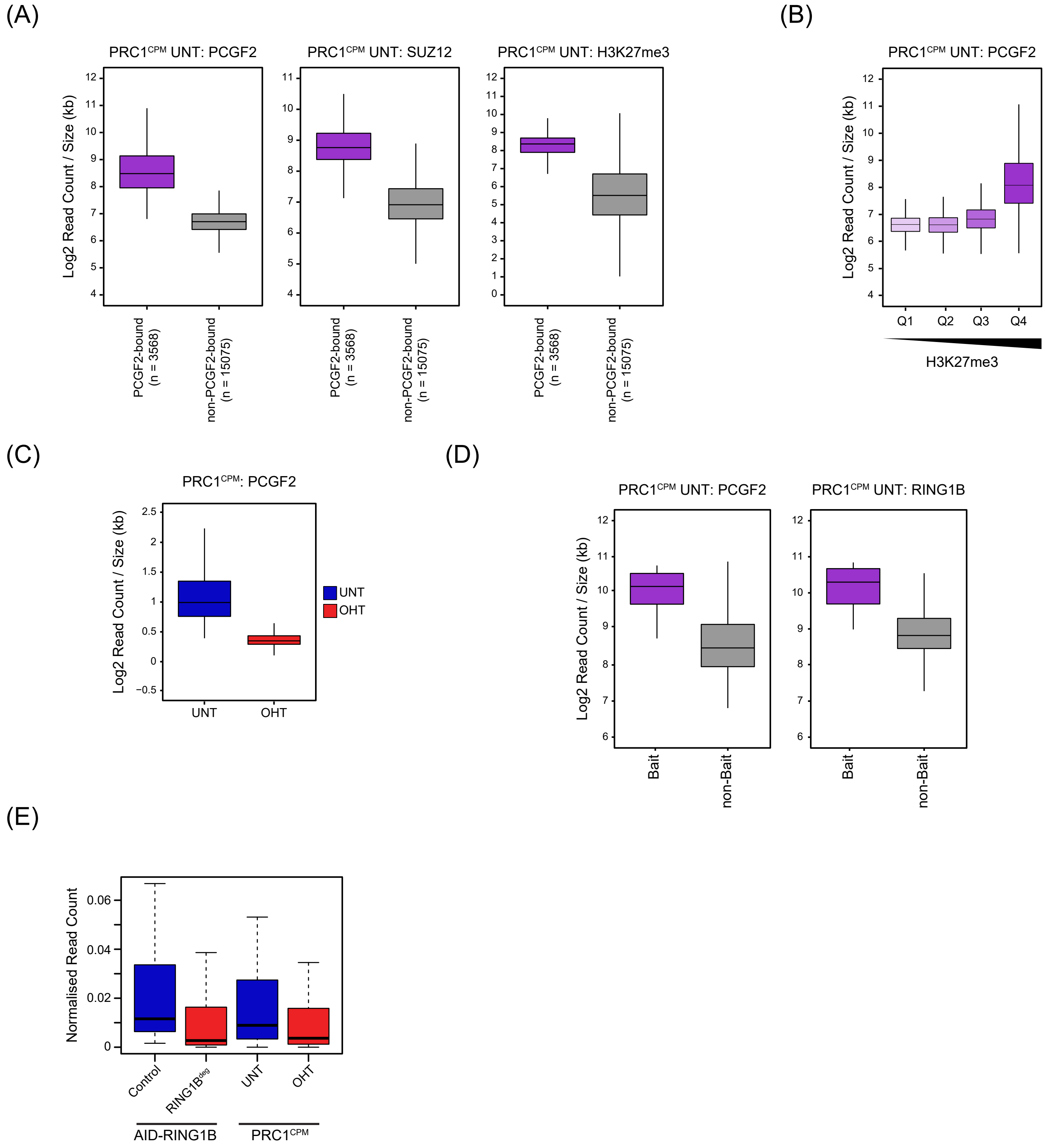

(A)
PRC1CPM UNT: PCGF2
PRC1CPM UNT: SUZ12
PRC1CPM UNT: H3K27me3
Log2 Read Count / Size (kb)
PCGF2-bound (n = 3568)
non-PCGF2-bound (n = 15075)
(B)
PRC1CPM UNT: PCGF2
Q1
Q2
Q3
Q4
H3K27me3
(C)
PRC1CPM: PCGF2
UNT
OHT
(D)
PRC1CPM UNT: PCGF2
PRC1CPM UNT: RING1B
Bait
non-Bait
(E)
Normalised Read Count
Control
RING1Bdeg
UNT
OHT
AID-RING1B
PRC1CPM

Figure S4

Figure S4. Related to Figure 4.

- **(A)** Box plots of the cChIP-seq signal for PCGF2, SUZ12 and H3K27me3 in untreated PRC1^{CPM} cells at RING1B-bound sites divided into sites that are bound (PCGF2 target sites) or not bound by PCGF2.
- **(B)** Box plots comparing the cChIP-seq signal for PCGF2 in untreated PRC1^{CPM} cells at RING1B-bound sites divided into quartiles (from lowest Q1 to highest Q4) based on H3K27me3 levels.
- **(C)** Box plots of the normalised cChIP-seq signal for PCGF2 at PCGF2 target sites in untreated and OHT-treated PRC1^{CPM} ESCs.
- **(D)** Box plots comparing PCGF2 and RING1B cChIP-seq signal in untreated PRC1^{CPM} cells at PCGF2 target sites used as baits in CaptureC analysis and the remaining PCGF2 target sites.
- **(E)** Box plot analysis of the normalised mean read count from CaptureC for the interactions between the bait Polycomb target regions and other PCGF2 target sites (n = 130) in auxin-treated *Ring1a*$^{-/-}$;AID-RING1B (RING1B^{deg}) ESCs (relative to the Control cell line with intact PRC1) and PRC1^{CPM} cells.

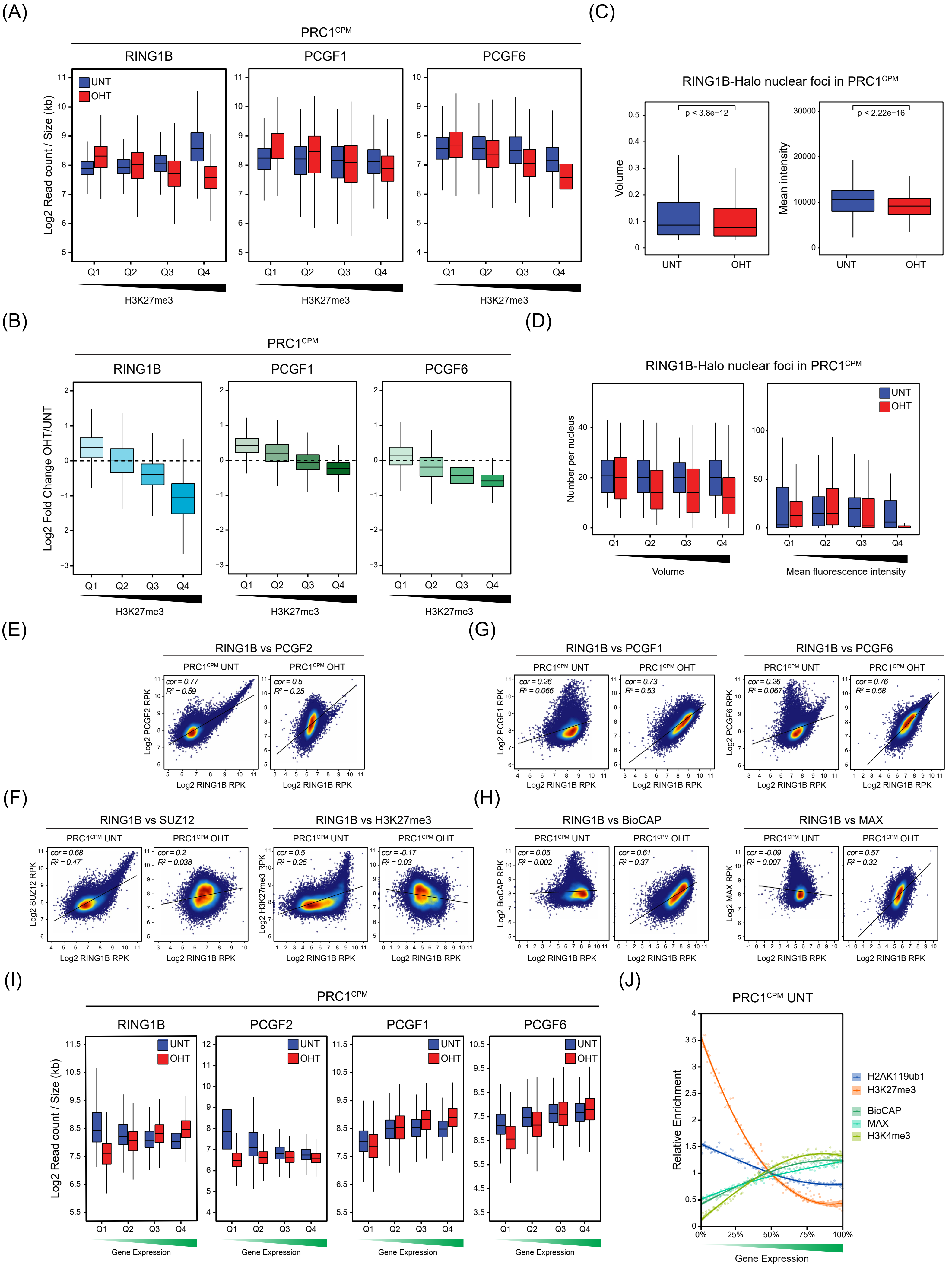

(A)
PRC1CPM
RING1B
PCGF1
PCGF6
UNT
OHT
Log2 Read count / Size (kb)
Q1 Q2 Q3 Q4
H3K27me3
(B)
Log2 Fold Change OHT/UNT
(C)
RING1B-Halo nuclear foci in PRC1CPM
p < 3.8e−12
p < 2.22e−16
Volume
Mean intensity
(D)
Number per nucleus
Volume
Mean fluorescence intensity
(E)
RING1B vs PCGF2
PRC1CPM UNT
PRC1CPM OHT
cor = 0.77
R² = 0.59
cor = 0.5
R² = 0.25
Log2 PCGF2 RPK
Log2 RING1B RPK
(F)
RING1B vs SUZ12
cor = 0.68
R² = 0.47
cor = 0.2
R² = 0.038
Log2 SUZ12 RPK
RING1B vs H3K27me3
cor = 0.5
R² = 0.25
cor = -0.17
R² = 0.03
Log2 H3K27me3 RPK
(G)
RING1B vs PCGF1
cor = 0.26
R² = 0.066
cor = 0.73
R² = 0.53
Log2 PCGF1 RPK
RING1B vs PCGF6
cor = 0.26
R² = 0.067
cor = 0.76
R² = 0.58
Log2 PCGF6 RPK
(H)
RING1B vs BioCAP
cor = 0.05
R² = 0.002
cor = 0.61
R² = 0.37
Log2 BioCAP RPK
RING1B vs MAX
cor = -0.09
R² = 0.007
cor = 0.57
R² = 0.32
Log2 MAX RPK
(I)
PCGF2
Gene Expression
(J)
PRC1CPM UNT
Relative Enrichment
H2AK119ub1
H3K27me3
BioCAP
MAX
H3K4me3
0% 25% 50% 75% 100%

Figure S5

Figure S5. Related to Figure 5.

- **(A)** Box plots of cChIP-seq signal for RING1B, PCGF1 and PCGF6 at RING1B-bound sites divided into quartiles based on H3K27me3 levels in untreated cells (from lowest Q1 to highest Q4) in $PRC1^{CPM}$ ESCs before and after OHT treatment. This shows that PCGF1 and PCGF6 occupancy is largely uniform across all RING1B-bounds sites with different H3K27me3 levels.
- **(B)** Box plots comparing changes in cChIP-seq signal for RING1B, PCGF1 and PCGF6 following OHT treatment in $PRC1^{CPM}$ cells at RING1B-bound sites divided into quartiles based on H3K27me3 levels in untreated cells. Together with (A), this shows that following removal of PRC1 catalytic activity, RING1B occupancy is reduced at sites with high levels of H3K27me3 (Q3 and Q4) but is retained/increased at sites with low levels of H3K27me3 (Q1 and Q2). In addition, it demonstrates that PCGF1 and PCGF6 occupancy is only modestly affected following loss of PRC1 catalytic activity, with binding of PCGF1 slightly increased at sites with low level of H3K27me3 and binding of PCGF6 moderately reduced at sites with high enrichment of H3K27me3.
- **(C)** Box plots comparing volumes and mean intensities of RING1B-Halo-JF_{549} nuclear foci in $PRC1^{CPM}$ ESCs before (n_{cells} = 69) and after OHT treatment (n_{cells} = 83). P-values denote statistical significance calculated by a Wilcoxon rank sum test.
- **(D)** Box plots comparing numbers of RING1B-Halo-JF_{549} nuclear foci detected in $PRC1^{CPM}$ ESCs before (n_{cells} = 69) and after OHT treatment (n_{cells} = 83), with foci divided into quartiles based on focus volume (*left*) or mean intensities (*right*) in untreated cells (from lowest Q1 to highest Q4). This shows that loss of PRC1 catalysis had the strongest effect on RING1B nuclear foci with the largest volume and mean intensity, while the foci with low to moderate intensities/volumes remained largely unchanged or even increased. This is in agreement with changes in RING1B occupancy by cChIP-seq at target sites with different levels of RING1B binding in untreated cells.
- **(E)** Scatter plots showing the relationship between the cChIP-seq signals of RING1B and PCGF2 at RING1B-bound sites in $PRC1^{CPM}$ ESCs before (UNT) and after OHT treatment (OHT). R^2 represents coefficient of determination for linear regression and *cor* denotes Pearson correlation coefficient.
- **(F)** As in (E) for RING1B and SUZ12 cChIP-seq (*left*) and RING1B and H3K27me3 cChIP-seq (*right*).
- **(G)** As in (E) for RING1B and PCGF1 cChIP-seq (*left*) and RING1B and PCGF6 cChIP-seq (*right*).
- **(H)** Scatter plots showing the relationship between the cChIP-seq signal for RING1B at RING1B-bound sites in $PRC1^{CPM}$ ESCs before (UNT) and after OHT treatment (OHT) with BioCAP-seq (*left*) or MAX ChIP-seq signal (*right*) in wild-type ESCs. R^2 represents coefficient of determination for linear regression and *cor* denotes Pearson correlation coefficient.
- **(I)** Box plots of RING1B, PCGF2, PCGF1 and PCGF6 cChIP-seq signal in untreated (UNT, blue) and OHT-treated (OHT, red) $PRC1^{CPM}$ ESCs at promoter-proximal RING1B-bound sites divided into quartiles based on the expression level of the associated gene in untreated cells (from lowest Q1 to highest Q4).
- **(J)** Relative enrichment of H2AK119ub1 and H3K27me3 cChIP-seq signal in untreated $PRC1^{CPM}$ ESCs and H3K4me3 and MAX ChIP-seq, as well as BioCAP-seq signal in wild-type ESCs across promoter-proximal RING1B-bound sites divided into percentiles based on the expression level of the associated gene in untreated $PRC1^{CPM}$ cells. For each factor, enrichment is shown relative to the fiftieth percentile. Lines represent smoothed conditional means based on loess local regression fitting.

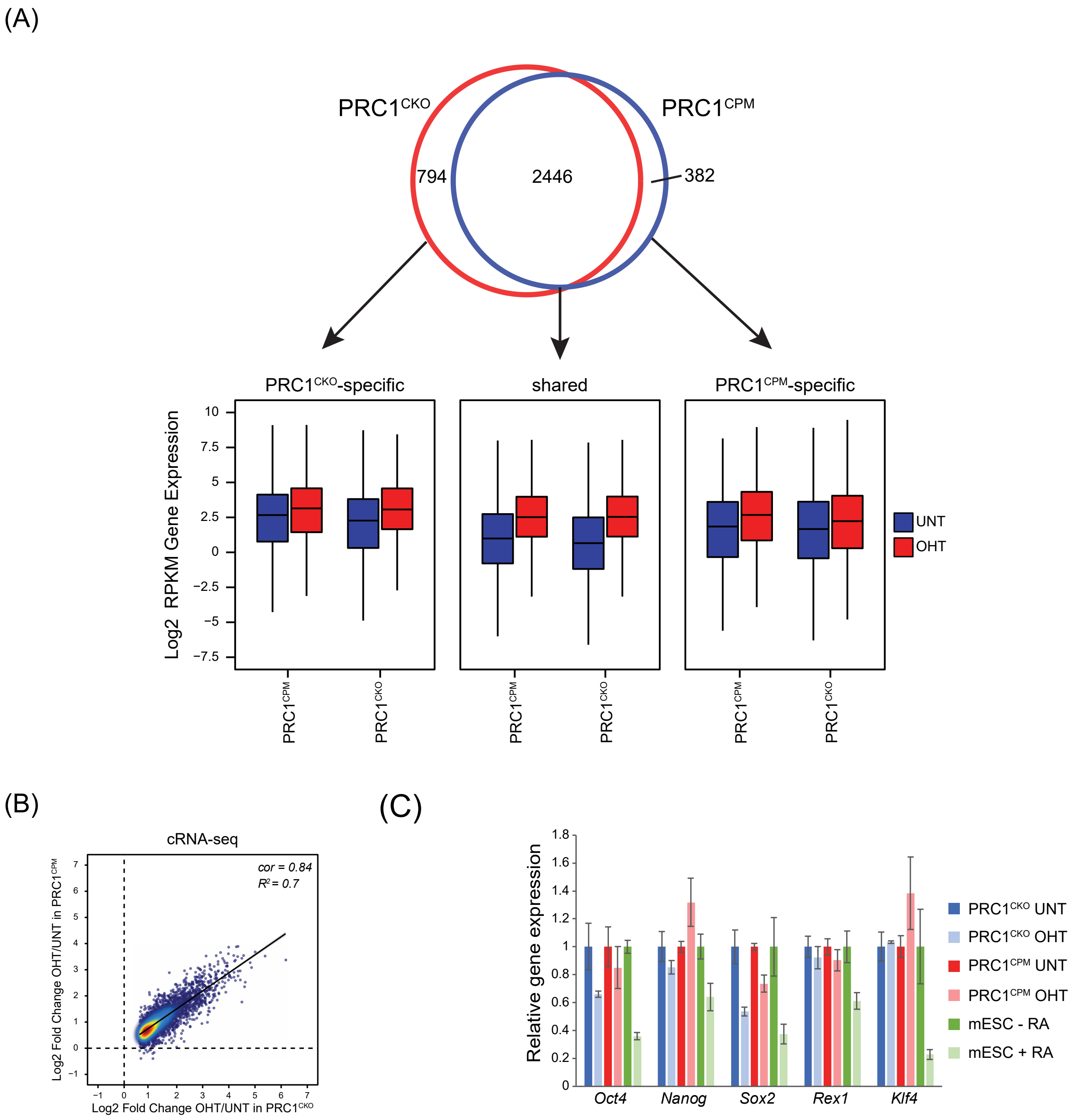

(A)
PRC1CKO
PRC1CPM
794
2446
382
PRC1CKO-specific
shared
PRC1CPM-specific
Log2 RPKM Gene Expression
10
7.5
5
2.5
0
−2.5
−5
−7.5
UNT
OHT
PRC1CPM
PRC1CKO
(B)
cRNA-seq
cor = 0.84
R2 = 0.7
Log2 Fold Change OHT/UNT in PRC1CPM
Log2 Fold Change OHT/UNT in PRC1CKO
(C)
Relative gene expression
1.8
1.6
1.4
1.2
1
0.8
0.6
0.4
0.2
0
Oct4
Nanog
Sox2
Rex1
Klf4
PRC1CKO UNT
PRC1CKO OHT
PRC1CPM UNT
PRC1CPM OHT
mESC - RA
mESC + RA

Figure S6

Figure S6. Related to Figure 6.

- **(A)** *Top panel:* A Venn diagram showing the overlap between the genes derepressed (p-adj < 0.05 and > 1.5-fold) in $PRC1^{CKO}$ (red) and $PRC1^{CPM}$ (blue) ESCs following OHT treatment.
  Bottom panel: Box plots comparing the gene expression levels from cRNA-seq in $PRC1^{CPM}$ and $PRC1^{CKO}$ ESCs before (UNT) and after OHT treatment (OHT) for three groups of derepressed genes defined in the Venn diagram above: $PRC1^{CKO}$-specific, shared between $PRC1^{CKO}$ and $PRC1^{CPM}$ ESCs, and $PRC1^{CPM}$-specific. This demonstrates that for the genes categorised as specifically derepressed in either $PRC1^{CKO}$ or $PRC1^{CPM}$ OHT-treated ESCs, the magnitude of gene expression changes is more modest than for the shared targets, making them more sensitive to the choice of a significance threshold. Importantly, expression levels of these genes following OHT treatment are highly similar between the two cell lines.
- **(B)** A scatter plot comparing the log2 fold changes in gene expression in cRNA-seq following OHT treatment in $PRC1^{CPM}$ and $PRC1^{CKO}$ ESCs for PRC1-repressed genes. R^2 represents coefficient of determination for linear regression and *cor* denotes Pearson correlation coefficient.
- **(C)** Expression of key pluripotency-associated genes in $PRC1^{CKO}$ and $PRC1^{CPM}$ cells, as well as in ESCs before (-RA) and after 72 hr retinoic acid treatment (+RA) to induce differentiation. Read counts from cRNA-seq ($PRC1^{CKO}$ and $PRC1^{CPM}$) or 4sU RNA-seq (-RA and +RA ESCs (Dimitrova et al., 2018)) were normalised to gene expression in the corresponding untreated cells. Error bars show SEM (n=3).

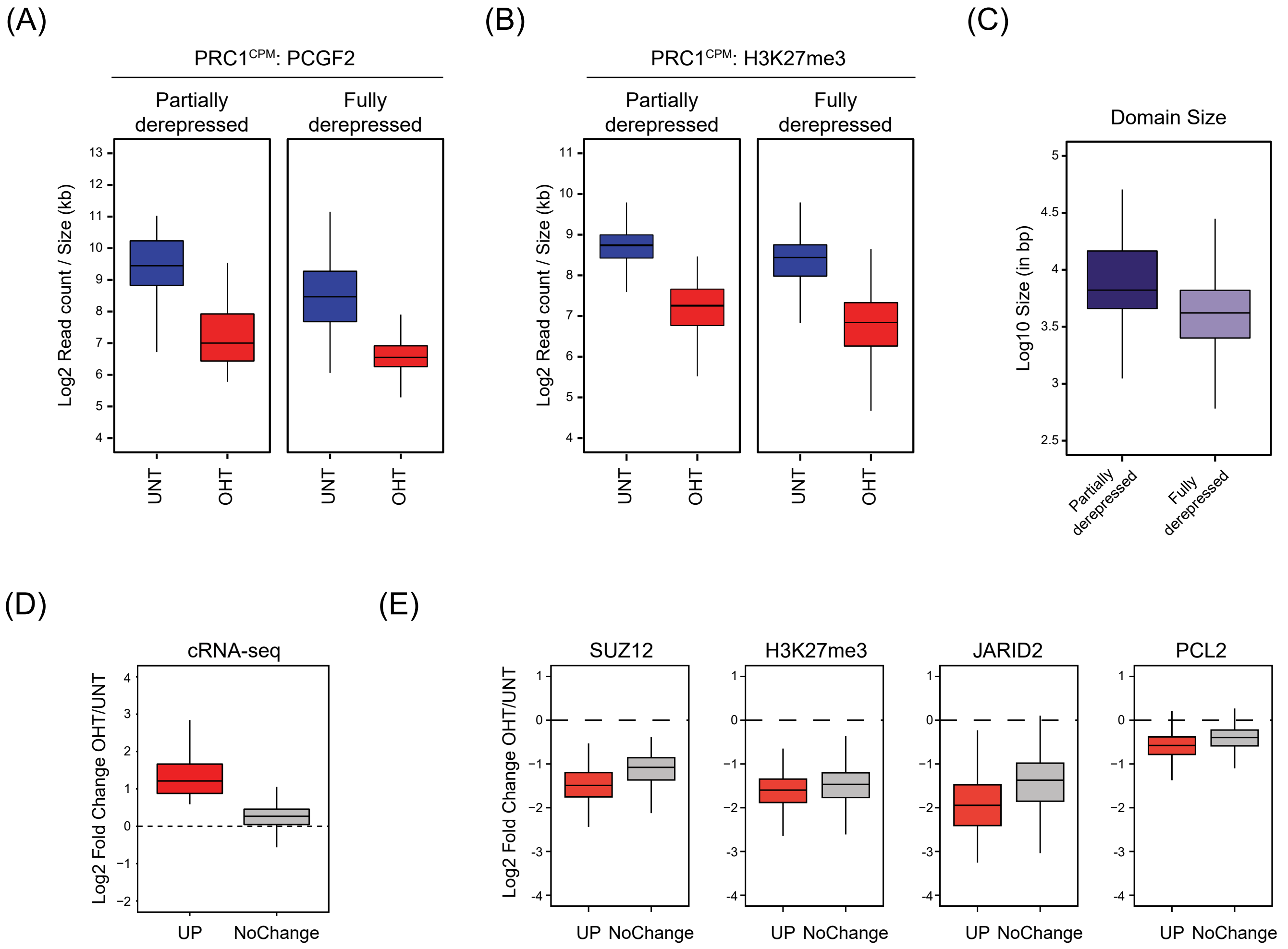

(A)
PRC1CPM: PCGF2
Partially derepressed
Fully derepressed
Log2 Read count / Size (kb)
UNT
OHT
(B)
PRC1CPM: H3K27me3
(C)
Domain Size
Log10 Size (in bp)
(D)
cRNA-seq
Log2 Fold Change OHT/UNT
UP
NoChange
(E)
SUZ12
H3K27me3
JARID2
PCL2

Figure S7

Figure S7. Related to Figure 7.

- **(A)** Box plots of PCGF2 cChIP-seq signal in $PRC1^{CPM}$ cells before (UNT) and after OHT treatment (OHT) at RING1B-bound sites associated with the promoters of PRC1-repressed genes which are either partially (n = 241) or fully derepressed (n = 2241) following loss of PRC1 catalysis as compared to a complete loss of PRC1.
- **(B)** As in (A) for H3K27me3 cChIP-seq.
- **(C)** Box plots comparing sizes of RING1B-bound sites overlapping with the promoters of PRC1-repressed genes which are either partially (n = 241) or fully derepressed (n = 2241) following OHT treatment in $PRC1^{CPM}$ as compared to $PRC1^{CKO}$ ESCs.
- **(D)** Box plots of log2 fold expression changes (cRNA-seq) for genes associated with PcG-occupied sites which show a significant reduction in SUZ12 levels following OHT treatment in $PRC1^{CPM}$ cells, divided into genes that become derepressed (p-adj < 0.05 and > 1.5-fold) following loss of PRC1 catalysis (UP, n = 1766) and those that do not change in expression (NoChange, n = 1779).
- **(E)** Box plots of log2 fold changes in PRC2 (SUZ12, JARID2 and PCL2) and H3K27me3 cChIP-seq signal at promoter-proximal PcG-occupied sites which show a significant reduction in SUZ12 levels following OHT treatment in $PRC1^{CPM}$ cells, divided into sites which are associated with genes that become derepressed (p-adj < 0.05 and > 1.5-fold) following loss of PRC1 catalysis (UP, n = 1766) and those that do not change in expression (NoChange, n = 1779).
